# Proteomic profiling identifies novel inflammation-related plasma proteins associated with ischemic stroke outcome

**DOI:** 10.1186/s12974-023-02912-9

**Published:** 2023-10-04

**Authors:** Annelie Angerfors, Cecilia Brännmark, Cecilia Lagging, Kara Tai, Robert Månsby Svedberg, Björn Andersson, Christina Jern, Tara M. Stanne

**Affiliations:** 1 Institute of Biomedicine, Department of Laboratory Medicine, Sahlgrenska Academy, University of Gothenburg, Gothenburg, Sweden; 2 Department of Research, Development, Education and Innovation, Region Västra Götaland, Sahlgrenska University Hospital, Gothenburg, Sweden; 3 Department of Clinical Genetics and Genomics, Region Västra Götaland, Sahlgrenska University Hospital, Gothenburg, Sweden; 4 Bioinformatics and Data Center, Core Facilities, Sahlgrenska Academy, University of Gothenburg, Gothenburg, Sweden

**Keywords:** Ischemic stroke, Proteomics, TNFSF14 [LIGHT] protein, TNFSF10 [TRAIL] protein, Oncostatin M protein, S100A12 protein, Sirtuin 2 protein, STAM-binding protein, CSF-1, Brain ischemia, Stroke

## Abstract

**Background:**

The inflammatory response to cerebral ischemia is complex; however, most clinical studies of stroke outcome focus on a few selected proteins. We, therefore, aimed to profile a broad range of inflammation-related proteins to: identify proteins associated with ischemic stroke outcome that are independent of established clinical predictors; identify proteins subsets for outcome prediction; and perform sex and etiological subtype stratified analyses.

**Methods:**

Acute-phase plasma levels of 65 inflammation-related proteins were measured in 534 ischemic stroke cases. Logistic regression was used to estimate associations to unfavorable 3-month functional outcome (modified Rankin Scale score > 2) and LASSO regressions to identify proteins with independent effects.

**Results:**

Twenty proteins were associated with outcome in univariable models after correction for multiple testing (FDR < 0.05), and for 5 the association was independent of clinical variables, including stroke severity (TNFSF14 [LIGHT], OSM, SIRT2, STAMBP, and 4E-BP1). LASSO identified 9 proteins that could best separate favorable and unfavorable outcome with a predicted diagnostic accuracy (AUC) of 0.81; three associated with favorable (CCL25, TRAIL [TNFSF10], and Flt3L) and 6 with unfavorable outcome (CSF-1, EN-RAGE [S100A12], HGF, IL-6, OSM, and TNFSF14). Finally, we identified sex- and etiologic subtype-specific associations with the best discriminative ability achieved for cardioembolic, followed by cryptogenic stroke.

**Conclusions:**

We identified candidate blood-based protein biomarkers for post-stroke functional outcome involved in, e.g., NLRP3 inflammasome regulation and signaling pathways, such as TNF, JAK/STAT, MAPK, and NF-κB. These proteins warrant further study for stroke outcome prediction as well as investigations into the putative causal role for stroke outcome.

**Supplementary Information:**

The online version contains supplementary material available at 10.1186/s12974-023-02912-9.

## Introduction

There are great inter-individual variations in outcomes after ischemic stroke. While this can in part be explained by clinical factors, acute interventions, and level of rehabilitation received [1], there is a need to better understand the underlying molecular mechanisms involved in brain injury and recovery. Experimental studies convincingly show that cerebral ischemia triggers an inflammatory response involving a complex network of signaling pathways [2–4]; however, many immune-modulating proteins remain understudied in clinical ischemic stroke cohorts. Exploratory searches for such proteins associated with ischemic stroke outcomes in human studies could point towards candidate biomarkers meriting further investigation for outcome prediction models.

The inflammatory response after ischemic stroke is a multistep pathway that initially involves Toll-like and Nod-like receptor (i.e., inflammasome) signaling followed by subsequent transcriptional activation of inflammatory mediators [2, 3]. The response includes resident brain cells as well as infiltrating peripheral immune cells due to blood–brain barrier disruption [2, 4, 5]. These stimulated receptors trigger intracellular signaling cascades (e.g., JAK/STAT, MAPK/ERK, NF-κB, and mTOR) [2], which in turn can rapidly affect expression of a variety of genes involved in the inflammatory and immune responses (e.g., cytokines and chemokines) [4–6]. Inflammation can have a paradoxical role in the response to cerebral ischemia. It can be neurotoxic and exacerbate the initial injury, contribute to expansion of the infarct and the development of cerebral edema. On the other hand, it can also be neuroprotective and promote repair by influencing infarct resolution and remodeling [4, 6, 7].

With regard to clinical studies of circulating levels of pro-inflammatory biomarkers in acute ischemic stroke, interleukin (IL)-6 is the most extensively studied. IL-6 stimulates hepatic expression of the downstream marker C-reactive protein (CRP), and elevated circulating levels of both proteins have been associated with increased stroke severity and unfavorable functional outcome [8]. Examples of other mediators more upstream in the inflammatory cascade that have been studied with respect to stroke outcomes include pro-inflammatory proteins, such as interleukin-1 beta (IL-1β), interferon-gamma (IFN-γ) and tumor necrosis factor-alpha (TNF-α) [9, 10], as well as anti-inflammatory proteins, such as IL-10, IL-4 and TNF-β [3]. Given the intricacy and multidimensionality of this response, other cytokines and immune-modulating proteins more directly coupled to underlying mechanisms and signaling pathways are likely also involved. However, for the most part, clinical studies examining their relationship to post-stroke functional outcome remain scarce.

We recently profiled a broad range of inflammation-related proteins (including chemokines, interleukins, surface molecules, and immune receptors) in relation to case control status in the *Sahlgrenska Academy Study on Ischemic Stroke* (*SAHLSIS*) and found many to be associated with ischemic stroke and the main etiological subtypes [11]. Here, we leverage this data in an exploratory study with the primary aim to determine whether acute-phase levels of any of these inflammation-related proteins are associated with 3-month functional outcome ischemic stroke, and to assess whether these associations are independent of established clinical predictors of stroke outcome. A secondary aim was to identify the proteins contributing most to the separation between favorable and unfavorable outcome, and to test their predictive accuracy. In addition to all ischemic stroke, we perform sex- and etiological subtype stratified analyses.

## Methods

Our study fulfills the STROBE checklist guidelines.

### Study population

The *Sahlgrenska Academy Study on Ischemic Stroke* (*SAHLSIS*), is a hospital-based observational, longitudinal cohort study which has been described in detail elsewhere [12]. In brief, 600 patients with a first-ever or recurrent acute ischemic stroke at 18–69 years of age were consecutively recruited at four different stroke units in Western Sweden between 1998 and 2003 [12]. Acute ischemic stroke was defined as rapidly developing clinical signs of focal disturbance of cerebral function lasting more than 24 h without hemorrhage or signs of another cause on neuroimaging. All patients included in this study underwent imaging of the brain (computed tomography and/or magnetic resonance) as part of the clinical routine investigation. If clinically indicated, patients were also evaluated by angiography and perfusion measures. The patients underwent additional work-up according to national guidelines, and patients were excluded if further evaluation showed another etiology than stroke. Information regarding hypertension, smoking, diabetes mellitus, hyperlipidemia and medications was registered at the 3-month follow-up by examinations and a structured questionnaire, as described [12]. Signs of infection were identified by review of medical records as described [11].

### Stroke subtypes and recurrent events

Etiologic subtypes of ischemic stroke were classified according to the Trial of Org 10172 in Acute Stroke Treatment (TOAST) criteria with minor modifications as described [13, 14] into large artery atherosclerosis (LAA), small artery occlusion (SAO), cardioembolic (CE) stroke, cryptogenic stroke (defined here as no identified cause despite a complete evaluation), cervical arterial dissection, other determined cause, and undetermined stroke (defined either as incomplete evaluation or two or more causes identified). Recurrent strokes and coronary events (defined as a myocardial infarction, hospitalization for unstable angina, acute coronary artery bypass grafting or percutaneous coronary intervention) before the 3-month follow-up were identified by review of the medical records.

### Stroke severity and functional outcome

Maximum stroke severity within the first 7 days of admission to the hospital was scored using the Scandinavian Stroke Scale (SSS; missing scores for 3 patients) and converted to the National Institutes of Health Stroke Scale (NIHSS) using an established algorithm [15].

Functional outcome was assessed by the modified Rankin Scale (mRS) at an in person 3-month follow-up visit to a stroke neurologist (median 101 day post-stroke, interquartile range (IQR) 94–111 days) and dichotomized into favorable (mRS scores 0–2) vs unfavorable (mRS scores 3–6) outcome indicating independent vs dependent on others or dead. As the distribution of cases across each of the mRS score categories was skewed (mRS 0, *n* = 73; 1, *n* = 124; 2, *n* = 218; 3, *n* = 77; 4, *n* = 36; 5, *n* = 1; 6, *n* = 5), we did not have statistical power to also perform ordinal analysis of the mRS.

### Measurement of plasma levels of inflammation-related proteins and annotation

Blood sampling was performed in the morning within 10 days after index stroke (median 4 days, interquartile range (IQR) 3–6 days) after an overnight fast. Venous blood was collected in tubes containing 10% by volume ethylene–diamine–tetra-acetic acid (EDTA, Vacuette, Greiner Bio-One, Essen, Germany). Platelet depleted plasma was isolated within 2 h by centrifugation 2000 × *g* at 4 °C for 20 min, aliquoted, and stored at − 80 °C. Plasma levels of inflammation-related proteins were analyzed using the Proseek Multiplex Inflammation I panel as described by Olink Bioscience (Uppsala, Sweden) [16]. This panel measures 92 proteins with documented or suggested involvement in inflammatory processes or diseases. Plasma samples were randomly distributed across plates and all protein analyses were performed by board-certified laboratory technicians blinded to the clinical information.

Sixty-five proteins showed a pre-specified call rate of > 80% and were included in analyses, as described [11]. A list of all proteins with corresponding UniProt identities and alternative aliases analyzed in this study are provided (Additional file 1: Table S1). Normalization of data was performed in GenEx software using Olink Wizard providing Normalized Protein eXpression (NPX) data on a Log2-scale, where one-unit higher NPX value represents a doubling of protein level [16]. Limit of detection (LOD) was reported by Olink for each protein assay and data below the LOD were replaced by LOD/2 for each protein. Of the 65 proteins analyzed, 54 proteins had no samples and three proteins had one sample below the LOD (CCL28, FGF-23, and MCP-4). The remaining 8 proteins had the number of samples below the LOD as follows: IL-7, *n* = 3; AXIN1, *n* = 10; CASP-8, *n* = 12; EN-RAGE, *n* = 19; SLAMF1, *n* = 25; GDNF, *n* = 29; MCP-3, *n* = 82; and INF-gamma, *n* = 112.

The Gene Ontology (GO) knowledgebase (http://geneontology.org) was used to annotate biological process information [accessed May 2023].

### Missing data

Plasma samples were missing for 39 and data on functional outcome for 31 *SAHLSIS* participants. In total 534 participants (415 favorable and 119 unfavorable outcome) were included in the analyses.

### Statistical analyses

Comparisons in baseline characteristics between favorable and unfavorable outcome groups were made with χ^2^ test, ANOVA, and Student’s *t *test. Olink analyses were run in two batches and ComBat adjustment was used (R package sva) to remove variation due to batch effect. Correlations between proteins were assessed by Pearson’s correlation coefficients and hierarchical clustering was then performed to produce correlation plots.

Univariable binary logistic regression was used to estimate odds ratios and 95% confidence intervals ([ORs and 95% CI]) for the associations between individual protein levels (NPX values) and unfavorable functional outcome. Multivariable analyses were adjusted for age, sex, day of blood draw, and diabetes mellitus (model 1) and additionally for stroke severity (model 2; covariables as in model 1 and stroke severity). These variables were chosen as they are either known to directly and independently associate to functional outcome after ischemic stroke (age, stroke severity, and diabetes mellitus); or may directly influence protein levels (sex and day of blood draw). As the NPX value is given on a log2 scale, the yielded OR corresponds to the predicted increased odds of suffering an unfavorable outcome per each doubling of the protein levels. Multiple testing was controlled for with the Benjamini and Hochberg (false discovery rate [FDR]) method in R using the p.adjust command. Associations with FDR < 0.05 were considered statistically significant and associations with FDR ≥ 0.05 but *P* < 0.05 were considered suggestive.

Least absolute shrinkage and selection operator (LASSO) regression analysis was used to identify a subset of the 65 proteins that contributed the most to separation between outcome groups (R packages glmnet and caret). The optimal lambda value for each model was identified using repeated cross-validation (fivefold, repeated 20 times) and a grid search approach (lambda ranging from 0.0001 to 1) with a fixed alpha of 1. To assess the predictive accuracy of the LASSO selected proteins, we used receiver operating characteristic analysis to calculate the area under the curve (AUC) for the multiprotein model as well as the individual proteins contributing to this model both with and without stroke severity (R packages pROC and caret).

Univariable and LASSO regressions as well as AUCs for the resulting multiprotein models were repeated after stratification by sex as well as the 4 main TOAST subtypes LAA, SAO, CE and cryptogenic stroke. We also performed sensitivity analyses excluding cases with signs of infection (*n* = 18), recurrent vascular events before the 3-month follow-up (*n* = 32), lipid-lowering treatment (*n* = 67), and cases who died during follow-up (i.e., mRS score 6, *n* = 5). For all stratified and sensitivity analyses *P* < 0.05 were considered significant.

Statistical analyses were performed in IBM SPSS Statistics 26 or R version 4.1.3, and all tests were 2-tailed. The R packages ggplot2 and corrplot were used for visualization.

## Results

Baseline characteristics for the ischemic stroke cases are detailed in Table 1. Cases with unfavorable outcome had significantly higher NIHSS score (i.e., stroke severity), whereas no significant differences were observed for hypertension, hyperlipidemia or smoking (*P* > 0.1 for all)*.*

**Table 1:** Baseline characteristics of ischemic stroke cases in *SAHLSIS* for the whole study cohort, and for the favorable and unfavorable 3-month functional outcome groups

	Total	Favorable	Unfavorable
*n*	534	415	119
Age, median [IQR], years	58 [52–64]	58 [52–64]	59 [53–65]
Male sex, *n* (%)	340 (64)	258 (62)	82 (69)
Timing of blood draw, median [IQR], days after index stroke	4 [3–6]	4 [3–6]	4 [2–6]
Hypertension, *n* (%)	320 (60)	247 (59)	73 (63)
Diabetes mellitus, *n* (%)	100 (19)	73 (18)	27 (23)*
Hyperlipidemia, *n* (%)	379 (71)	295 (74)	84 (80)
Smoking, *n* (%)	208 (39)	164 (40)	44 (37)
Body mass index, median [IQR], kg/m^2^	26 [24–29]	26 [24–29]	26 [23–29]
Stroke severity, maximum NIHSS score, median [IQR]	3 [2–7]	2 [1–4]	12 [7–16]^†^
Etiological subtype of ischemic stroke (TOAST)			
Large artery atherosclerosis, *n* (%)	68 (12)	48 (11)	20 (15)*
Small artery occlusion, *n* (%)	115 (20)	104 (24)	11 (8)
Cardioembolic, *n* (%)	97 (17)	67 (15)	30 (23)
Cryptogenic, *n* (%)	154 (27)	122 (28)	32 (24)
Cervical arterial dissection, *n* (%)	30 (5)	17 (4)	13 (10)
Other determined, *n* (%)	17 (3)	12 (3)	5 (4)
Undetermined, *n* (%)	88 (15)	68 (15)	20 (15)
Stratified analyses (*n*)			
Sex, males vs females	340 vs 194	258 vs 157	82 vs 37
Sensitivity analyses (*n*)			
Excluding *n* = 16 cases with signs of infection	518	408	110
Excluding *n* = 23 cases with recurrent stroke/other major vascular event	511	403	108
Excluding *n* = 67 with lipid-lowering treatment	467	365	102

Difference between favorable and unfavorable outcome were tested by χ^2^ test, ANOVA, and Student’s *t *test. *P < 0.1, ^†^*P* < 0.001

### Associations of individual proteins with 3-month functional outcome

**Fig. 1:**
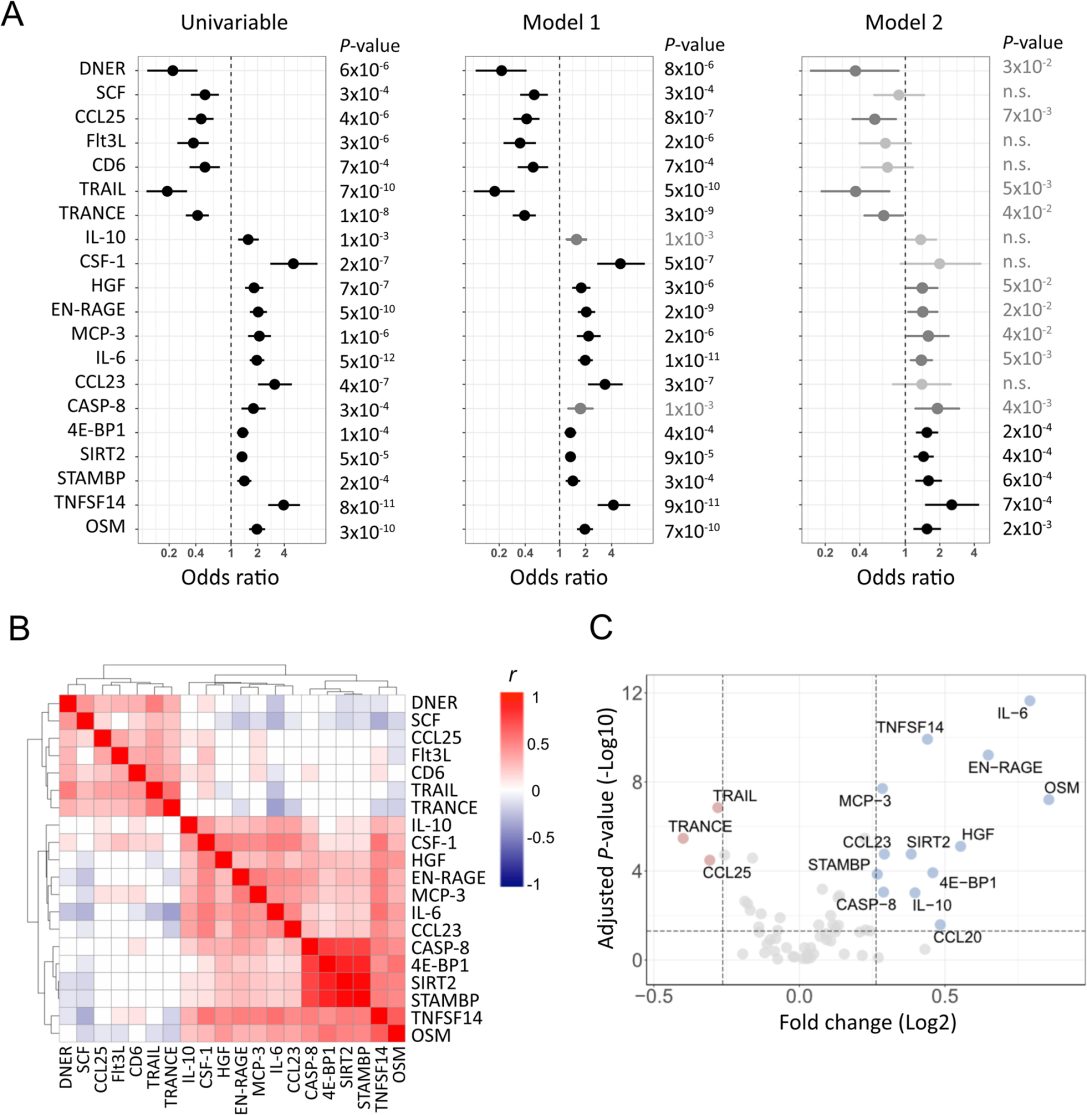
Associations between acute-phase plasma protein levels and 3-month functional outcome in 534 ischemic stroke cases. **A** Odds ratios and 95% confidence intervals for unfavorable outcome per doubling of protein levels as compared to favorable outcome. Associations with false discovery rate (FDR) < 0.05 are marked in bold and black, and suggestive associations (FDR ≥ 0.05 and *P* < 0.05) in grey. **B** Hierarchical protein clusters based on Euclidean distance. Pearson’s correlation coefficients (r) between proteins are displayed, where correlations with *P* < 0.05 are marked in colour. Positive correlations, red; and inverse correlations, blue. **C** Volcano plots showing fold-change in proteins between the favorable and unfavorable outcome groups. Blue circles, proteins with > 20% higher levels in unfavorable vs favorable outcome; red circles, proteins with < 20% lower levels in unfavorable outcome; grey circles, no significant fold-change. Protein names are provided in Additional file 1: Table S1

In univariable analyses 20 proteins were significantly associated with outcome (FDR < 0.05), as depicted in Fig. 1A. Among them, CSF-1 and TNFSF14 had the largest ORs with an approximately fourfold higher odds of unfavorable outcome per doubling in plasma levels; and TRAIL and DNER had the smallest ORs with ~ threefold lower odds of unfavorable outcome. Effect sizes and significance levels for all 65 proteins, including 20 proteins with suggestive association to outcome (FDR ≥ 0.05 and *P* < 0.05), are available in Additional file 1: Table S2.

Results for the 20 proteins with significant association to outcome are displayed by hierarchical cluster order in Fig. 1B, which shows two distinct clusters of proteins. For the 13 proteins in the first cluster, higher levels increased the odds of unfavorable outcome. For the 7 proteins in the second cluster, higher protein levels decreased the odds of unfavorable outcome. Of these 20 proteins, 16 had a mean fold-difference > 20% between favorable and unfavorable outcome, and they are displayed in the Volcano plot (Fig. 1C).

In multivariable analyses adjusted for age, sex, day of blood draw, and diabetes mellitus (model 1); 18 proteins were significantly (FDR < 0.05) and 21 proteins were suggestively (FDR ≥ 0.05 and *P* < 0.05) associated with outcome (Fig. 1A and Additional file 1: Table S2). In a final multivariable regression model, stroke severity was also adjusted for (model 2; Fig. 1A). In this analysis 5 proteins were significantly associated (FDR < 0.05) and 12 were suggestively associated with outcome (Fig. 1A and Additional file 1: Table S2). The proteins with the largest effect sizes in multivariable analyses were TNFSF14 and TRAIL.

### Multiprotein models

We applied a machine learning approach, i.e., LASSO regression, to identify the proteins contributing the most to the separation between favorable and unfavorable outcome. Starting with the 65 proteins a total of 9 proteins were selected for all ischemic stroke (Table 2). For 3 of these proteins elevated plasma levels were associated with a more favorable outcome and for 6 elevated levels were associated with less favorable outcome. We next evaluated the diagnostic accuracy of the selected proteins for predicting functional outcome, and these 9 proteins together yielded an AUC of 0.81 [95% CI 0.76–0.86], which increased to 0.92 [0.90–0.95] when stroke severity was added. For comparison, the AUC for stroke severity alone was 0.91 [0.88–0.94]. Of note, the AUC for stroke severity in the present study is higher than most studies, where severity is typically scored at one timepoint (e.g., admission, 24 h after admission, or discharge). AUCs for the individual proteins contributing to the LASSO model are available in Additional file 1: Table S3.

**Table 2:** Proteins selected by LASSO regression to contribute the most to the separation between favorable and unfavorable outcome, listed in order of importance for all ischemic stroke as well as for sex and stroke subtype stratified analyses

Order	All ischemic stroke	Males	Females	LAA	SAO	CE	Crypt
1	TRAIL	TRAIL	Flt3L	IL-6	GDNF	TRAIL	EN-RAGE
2	EN-RAGE	IL-6	IL-10RB	EN-RAGE	TNFB	EN-RAGE	CD6
3	IL-6	OSM	EN-RAGE	–	–	MCP-3	CCL25
4	OSM	EN-RAGE	SCF	–	–	IL-10	TNFSF14
5	Flt3L	TNFSF14	CSF-1	–	–	SIRT2	MCP-3
6	TNFSF14	CCL25	GDNF	–	–	Flt3L	CASP-8
7	CSF-1	CSF-1	uPA	–	–	HGF	NT-3
8	CCL25	–	SLAMF1	–	–	GDNF	TRANCE
9	HGF	–	TNFSF14	–	–	IL-8	CDCP1
10	–	–	–	–	–	CCL4	–

LAA, large artery atherosclerosis; SAO, small artery occlusion; CE, cardioembolic stroke; Crypt, cryptogenic stroke. Protein names are provided in Additional file 1: Table S1

### Stratified and sensitivity analyses

**Fig. 2:**
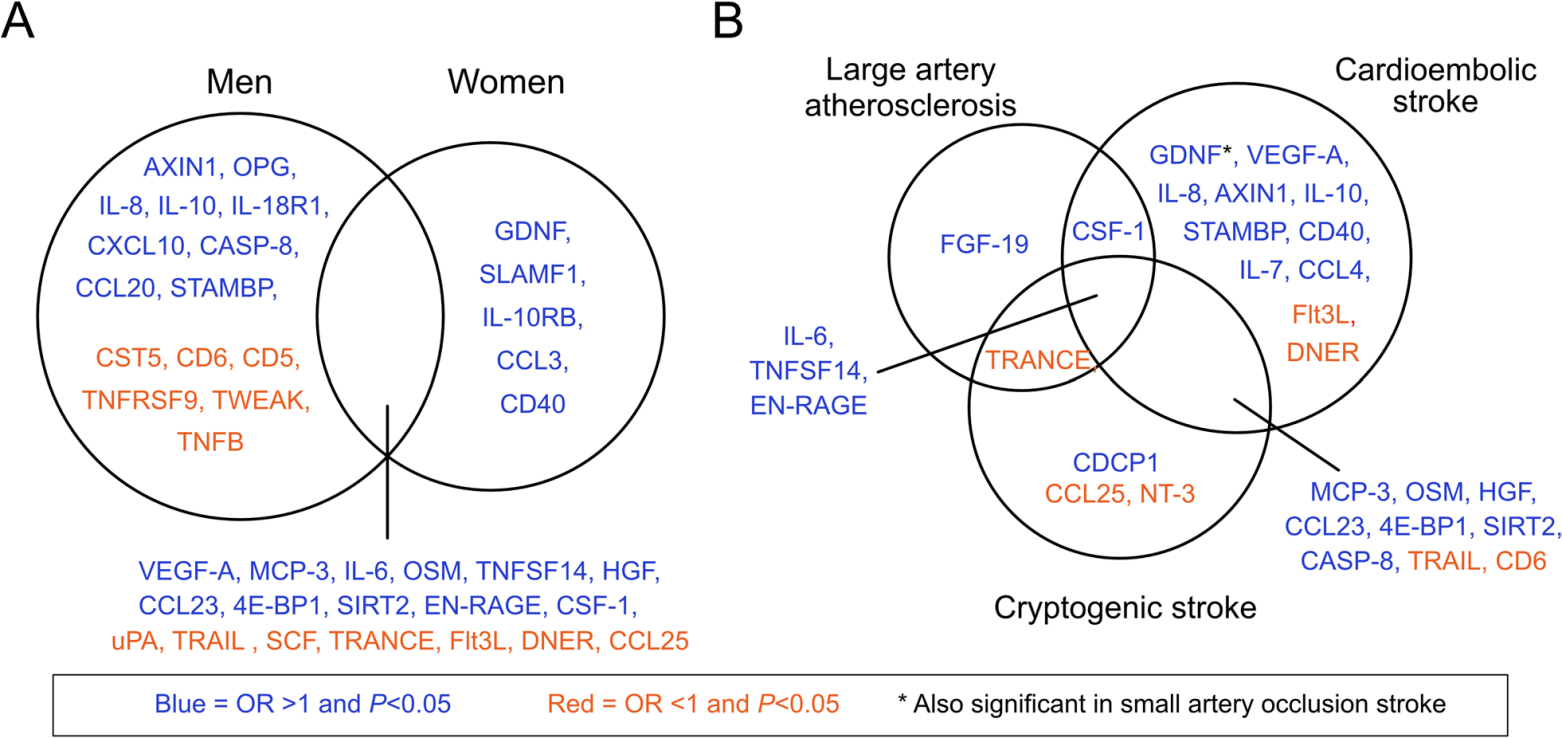
Venn diagram showing unique and shared proteins across patient strata. **A** Sex. **B** Etiological ischemic stroke subtype. Protein names are provided in Additional file 1: Table S1

Univariable regressions were repeated stratifying according to sex and TOAST subtype (effect sizes and significance levels for all 65 proteins are available in Additional file 1: Table S4). A total of 18 proteins were associated with outcome and directionally concordant in men and women (at *P* < 0.05). An additional 15 proteins were associated with outcome in men only, and 5 were associated with outcome in women only (Fig. 2A). In men and women, LASSO regression selected 7 and 9 proteins, respectively, that contributed to the separation between favorable and unfavorable outcome with an AUC of 0.82 [0.76–0.87] and 0.86 [0.79–0.93], that increased to 0.96 [0.91–1] and 0.95 [0.91–0.99], respectively, when stroke severity was added. For details of the LASSO selected proteins, see Table 2, and for AUCs of individual proteins see Additional file 1: Table S3.

When stratifying according to TOAST subtype, the highest number of proteins significantly associated with outcome were in the subtype CE stroke (24 proteins at *P* < 0.05), followed by cryptogenic stroke (16), LAA (6), and SAO stroke had the least (1 protein; Fig. 2B). LASSO regressions selected the following number of proteins that contributed to the separation between favorable and unfavorable outcome per subtype: LAA and SAO, 2 proteins each with AUC of 0.79 and 0.82, respectively (increasing to 0.91 for both when stroke severity was added); CE stroke 10 proteins with AUC of 0.93 (increasing to 0.97 when stroke severity was added); and cryptogenic stroke 9 proteins with AUC of 0.89 (increasing to 0.98 when stroke severity was added). For details of the LASSO selected proteins, see Table 2, and for AUCs of individual proteins see Additional file 1: Table S3. Due to the small sample size, especially for SAO stroke, these results should be interpreted with caution.

We also conducted sensitivity analyses to exclude cases (1) with clinical signs of infection at the time of blood draw, (2) who experienced a recurrent stroke or coronary event during follow-up, (3) on lipid-lowering treatment prior to admission, and (4) who died during follow-up. Although the effect size and significance levels of top findings were different in the various sensitivity analyses, the proteins with the largest and smallest ORs across sensitivity analyses were CSF-1 and TNFSF14 (ranging from 3.8 to 5.5); and TRAIL and DNER (ranging from 0.19 to 0.25), respectively (*P* < 1 × 10^–5^ for all; see Additional file 1: Table S5).

## Discussion

In this explorative study of 65 proteins implicated in inflammation we identified many candidate plasma protein biomarkers associated with 3-month functional outcome after ischemic stroke, and for 5 proteins the association was independent of established clinical predictors of outcome, including stroke severity. Furthermore, LASSO regression identified 9 proteins that could best separate favorable and unfavorable outcome with an estimated diagnostic accuracy of 0.81. Elevated plasma levels of 3 proteins were associated with a more favorable outcome and 6 with less favorable outcome, consistent with inflammation having both detrimental and protective effects after cerebral ischemia. Finally, we identified sex- and etiologic subtype-specific associations. This was reflected also in the LASSO regressions, wherein different subsets of proteins best separated outcomes in the different patient strata.

**Fig. 3:**
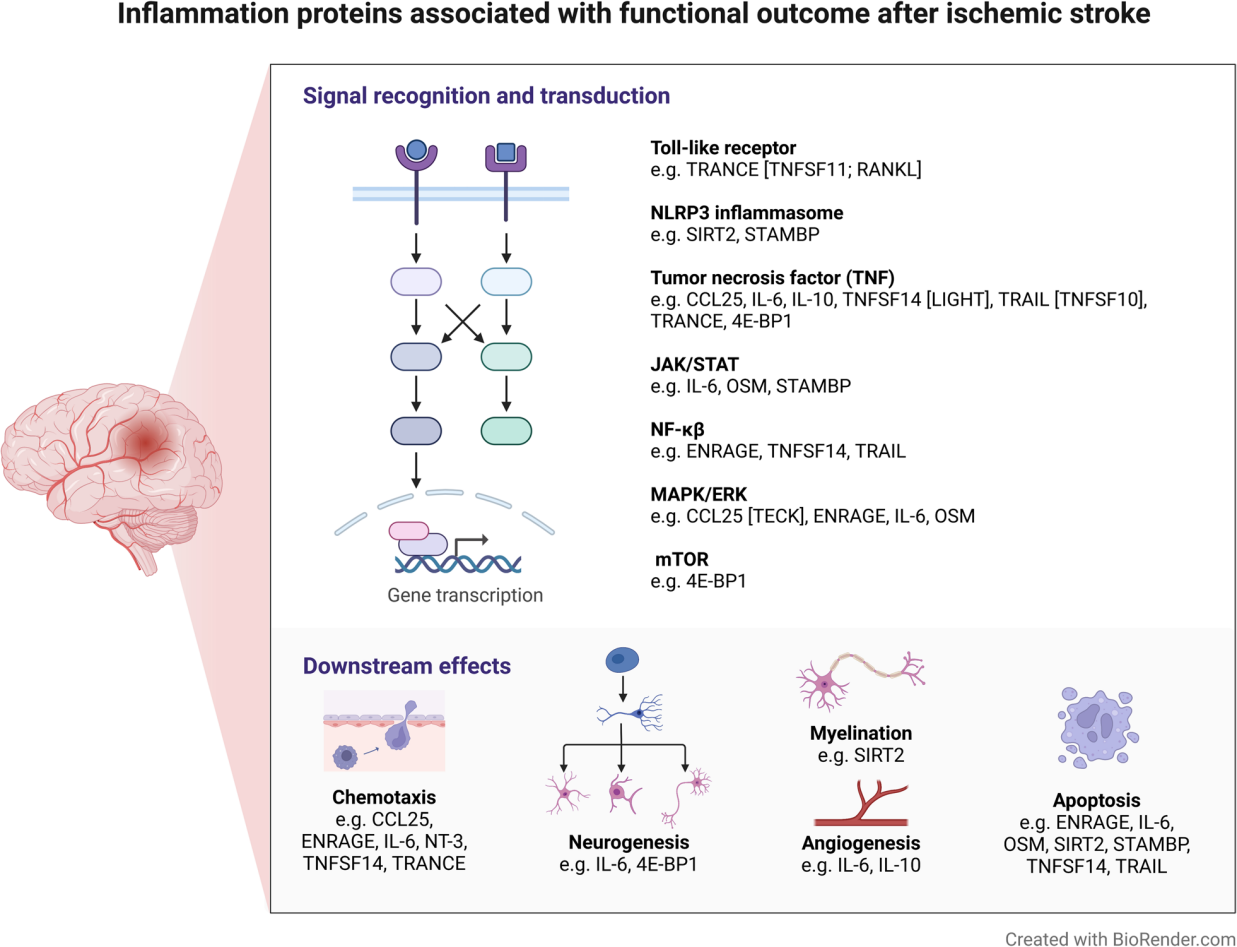
Selected gene ontology (GO) biological process classifications of the proteins associated with 3-month functional outcome after ischemic stroke. Protein names and a complete list of all GO terms are provided in Additional file 1: Table S1

Our study validates findings for several inflammatory mediators from clinical or experimental studies in relation to stroke outcomes. Elevated IL-6 was associated with unfavorable outcome, in line with previous clinical studies [8, 17]. However, it is of note that IL-6 did not withstand correction for stroke severity in this study. EN-RAGE is a member of the S100 family of calcium-binding proteins and promotes inflammation and apoptosis through MAPK/ERK and NF-κB signaling (Fig. 3). Our results are in validation of two small clinical studies, where elevated EN-RAGE plasma protein levels (*n* = 171) [18] and *S100A12* mRNA isolated from peripheral blood were associated with unfavorable 3-month functional outcome after stroke [19]. SIRT2 is an NAD + -dependent deacetylase highly expressed by both neurons and oligodendrocytes and has been implicated in regulating for instance apoptosis [20] and myelination (Fig. 3) and is a known regulator of the NLRP3 inflammasome [21] and MAPK signaling [22] (Fig. 3). In animal models of stroke, SIRT2 inhibition showed neuroprotective effects [22], which is directionally consistent with our findings. Our results are concordant with a recent small study of 75 ischemic stroke patients in whom higher SIRT2 in serum exosomes was associated with unfavorable 3-month functional outcome [23]. We also identified TRAIL, a cytokine that plays a role in the regulation of apoptosis [24, 25] (Fig. 3). In contrast to the proteins above, TRAIL has been shown to have neuroprotective effects in experimental models of cerebral ischemia [26]. This is analogous with what we observe here, where elevated TRAIL is associated with favorable outcome, and this is also in line with results for cardiovascular disease [25].

Notably, we additionally provide novel data for several proteins without previous experimental or clinical support for a role in stroke outcomes. TNFSF14 is a transmembrane protein produced by activated T cells [27] and promotes the production of pro-inflammatory cytokines by activating the NF-κB signaling pathway (Fig. 3). It has a role in the pathogenesis of several inflammatory diseases, including rheumatoid arthritis, multiple sclerosis, and inflammatory bowel disease [28, 29]. In these diseases, blocking the interaction between TNFSF14 and its receptor has been shown to reduce inflammation and improve disease outcomes [29]. While studies on ischemic stroke outcomes are lacking, increased TNFSF14 predicts major adverse cardiovascular events in patients with stable coronary artery disease [30], which is directionally concordant to what we observe here. 4EBP1 is a eukaryotic initiation factor 4E (eIF4E)-binding protein, which plays a key role in translational regulation driven by the mTOR (mammalian target of rapamycin) signaling pathway [31] (Fig. 3). The stimulation of mTOR signaling after ischemic brain injury has several functions of plausible relevance for neural repair, such as promoting neural stem cell/progenitor cell proliferation and differentiation, enhancing angiogenesis and synaptic plasticity [31] and targeting this pathway has been proposed as a promising therapeutic strategy for stroke [32]. CCL25 [alias TECK] is a chemokine that together with its receptor (CCR9) is involved in the regulation of immune cell migration and activation and plays a role in the development of T-cells. Decreased levels of CCL25 have been reported in a variety of inflammation-related diseases [33]. Here, for the first time, we report that elevated CCL25 levels are associated with favorable outcome after stroke. STAMBP [alias AMSH] is a deubiquitinating enzyme that is involved in the regulation of protein turnover and intracellular signaling (e.g., JAK/STAT pathway, Fig. 3). STAMBP is a negative regulator of the NALP7 and NLRP3 inflammasomes [34, 35], the latter of which has been identified as a potential therapeutic target to reduce pro-inflammatory stress after stroke [36]. CSF-1, known as macrophage colony-stimulating factor is a primary driver of macrophage expansion in atherosclerosis [37]. Recently, genetically predicted higher CSF-1 was shown to associate with higher risk of ischemic stroke in a Mendelian randomization study, indicating a plausible causal role [38]. Here, we found that elevated acute phase CSF-1 is also associated with unfavorable outcome.

Understanding differences in the inflammatory response during an acute ischemic stroke in different patient strata may help explain why certain subgroups of patients are at higher risk of experiencing unfavorable outcome and could provide insight into distinct mechanisms. We, therefore, performed analyses stratified for sex and TOAST subtype to identify putative sex- or etiology-specific biomarkers. In general, associations were directionally similar across strata with the largest number of significant associations being observed in men and in cardioembolic stroke. While the discriminative ability achieved in multiprotein models for men and women were similar, the range was wider for TOAST subtypes (highest AUC in cardioembolic followed by cryptogenic stroke) which may in part be attributable to sample sizes and initial stroke severity. Examples of strata-specific associations include elevated levels of 5 proteins associated with unfavorable outcome in women only. For GDNF [39], CCL3 [alias MIP-1α] [40], and CD40 [alias TNFSF5] [41] sex-differences have been observed in experimental or clinical studies. This was also reflected in the LASSO regressions, where GDNF, IL-10RB and SLAMF1 contributed to the separation of outcomes in women only. Other noteworthy protein associations include elevated CCL25, CD6, TRANCE and NT-3 associated with favorable and CDCP1 with unfavorable prognosis in cryptogenic stroke, all of which were selected by LASSO regression for cryptogenic stroke. Interestingly, TRANCE [alias TNFSF11, also known as RANKL] and its receptor (RANK) have been reported to attenuate ischemic brain injury through inhibition of Toll-like receptor 4 signaling [42]. Derivatives of RANKL already exist as therapeutic agents in bone remodeling [42] and novel partial agonists are also under exploration for stroke [43]. NT-3 is a member of the neurotrophin family of growth factors, which are important mediators of neuronal survival and regeneration, and one experimental study found that NT-3 improves recovery after cerebral ischemia [44], which is directionally analogous to our finding. As these analyses were performed on small subgroups, future stratified analyses in larger ischemic stroke cohorts are clearly warranted.

As discussed above, some of the proteins that showed significant association to functional outcome after ischemic stroke in the present study have clinical or experimental support that is directionally concordant with our findings indicating that they are involved in pathways of importance for ischemic cerebral injury or repair. Mendelian randomization studies are, however, needed to infer possible causal relationships to outcome. In this context, should a causal relationship be found, it is of note that inflammation is currently considered a prime target for developing new stroke therapies to improve outcomes after stroke [4, 6, 7]. As there are several immunomodulating drugs already approved for treatment of inflammation-related diseases (e.g., rheumatoid arthritis and inflammatory bowel disease) and neurological disorders (e.g., multiple sclerosis and Alzheimer’s disease), **t**here may be repurposing opportunities for stroke [7].

The principal strengths of the current study are the inclusion of consecutive and well-characterized ischemic stroke cases, and measurements of plasma proteins with a highly specific and sensitive multiplex method. There are several limitations that need to be considered. First, and as indicated above, although several proteins have previous clinical or experimental support as plausible candidates for stroke outcome, the present results need to be replicated in large cohorts. This is especially true for the stratified analyses, in which the sample sizes are limited. Second, as we measured plasma proteins using a multiplex assay, some important proteins (e.g., interferon gamma, IL-1, IL-4 and TNF-α) had a large proportion of samples below the detection limit. Third, in this exploratory study, protein levels were measured using a relative quantification, and thus direct comparisons to other studies using absolute quantification are not possible. In this context, while we provide preliminary AUC calculations for diagnostic accuracy of these proteins, the results need to be replicated with assays that enable determination of plasma protein concentration cut-offs. Fourth, it should be noted that the study participants of the present study are relatively young (< 70 years of age), and consequently a large proportion has mild stroke. This limits the generalizability to cohorts with later-onset ischemic stroke and more severe strokes. Finally, our results might not be extrapolated to populations of other ethnicities.

In conclusion, we identified multiple candidate plasma protein biomarkers of 3-month outcome after ischemic stroke involved in, e.g., NLRP3 inflammasome regulation and signaling pathways, such as TNF, JAK/STAT, MAPK, and NF-κB. Several of these proteins have experimental evidence indicating a role in injury or recovery after stroke in support of our findings. These proteins warrant further study for stroke outcome prediction as well as investigations into the putative causal role for stroke outcome.

### Supplementary Information

**Additional file 1.** Supplementary Tables S1–S5.

## Data Availability

Anonymized data supporting the conclusions of this article will be shared upon reasonable request from a qualified academic investigator provided data transfer agrees with EU legislation on the general data protection regulation (GDPR) and with decisions by the Ethical Review Board of Sweden and the University of Gothenburg, the latter which should be regulated in a data transfer agreement.

## Abbreviations

CCL25: C–C motif chemokine 25 [alias TEC protein tyrosine kinase, TECK]
CE: Cardioembolic stroke
CSF-1: Colony stimulating factor 1
EN-RAGE: Extracellular newly identified receptor for advanced glycation end-products binding protein [alias S100 calcium binding protein A12, S100A12]
FDR: False discovery rate
IL: Interleukin
JAK/STAT: Janus kinase/signal transducer and activator of transcription
LAA: Large artery atherosclerosis
MAPK/ERK: Mitogen-activated protein kinase/extracellular-*signal*-regulated kinase
mRS: Modified Rankin Scale
mTOR: Mammalian target of rapamycin
NF-κB: Nuclear factor kappa-light-chain-enhancer of activated B cells
NIHSS: National Institutes of Health stroke scale
NLRP3: NOD-like receptor protein 3
SAO: Small artery occlusion
TOAST: Trial of Org 10172 in Acute Stroke Treatment
TNF: Tumor necrosis factor
TNFSF14: TNF super family member 14 [alias homologous to lymphotoxin, exhibits inducible expression and competes with herpes simplex virus glycoprotein D for herpes virus entry mediator, a receptor expressed by T cells, LIGHT]
TRAIL: TNF-related apoptosis-inducing ligand [alias TNF super family member 10, TNFSF10]
4EBP1: Eukaryotic initiation factor 4E-binding protein 1
