# Supplementary material for: Proteomic profiling identifies novel inflammation-related plasma proteins associated with ischemic stroke outcome
Source: J Neuroinflammation. 2023 Oct 4;20:224. doi: 10.1186/s12974-023-02912-9 (PMC10548608; doi:10.1186/s12974-023-02912-9)
Supplement: Supplementary file 1 — Additional file 1. Supplementary Tables S1–S5. [file 12974_2023_2912_MOESM1_ESM.pdf]

SUPPLEMENTARY INFORMATION

Journal of Neuroinflammation

Proteomic profiling identifies novel inflammation-related plasma proteins associated with ischemic stroke outcome

Annelie Angerfors[1], MScEng; Cecilia Brännmark[1,2], MD, PhD; Cecilia Lagging[1,3], MD; Kara Tai[1], MSc; Robert Månsby Svedberg[1], MD; Björn Andersson[4], PhD; Christina Jern[1, 3*] MD, PhD; Tara M. Stanne[1,3*], PhD

[1]Institute of Biomedicine, Department of Laboratory Medicine, Sahlgrenska Academy, University of Gothenburg, Sweden
[2]Region Västra Götaland, Sahlgrenska University Hospital, Department of Research, Development, Education and Innovation
[3]Region Västra Götaland, Sahlgrenska University Hospital, Department of Clinical Genetics and Genomics, Gothenburg, Sweden
[4]Bioinformatics and Data Center, Core Facilities, Sahlgrenska Academy, University of Gothenburg, Gothenburg, Sweden

*Equal contribution

Corresponding author: Tara Stanne, PhD, Assoc. Prof.; E-mail: tara.stanne@gu.se

TABLES

Table S1: Proteins in the Olink Proseek Multiplex Inflammatory panel, Uniprot IDs and annotated biological processed (Gene Ontology; GO terms)

Category	Name	Abbreviation	Uniprot
**Interleukins**			
	Interleukin-6	IL-6	P05231
	Interleukin-7	IL-7	P13232
	Interleukin-8	IL-8	P10145
	Interleukin-10	IL-10	P22301
	Interleukin-10 receptor subunit beta	IL-10RB	Q08334
	Interleukin-12 subunit beta	IL-12B	P29460
	Interleukin-18	IL-18	Q14116
	Interleukin-18 receptor 1	IL-18R1	Q13478
**Chemokines**			
	C-C motif chemokine 3	CCL3	P10147
	C-C motif chemokine 4	CCL4	P13236
	C-C motif chemokine 19	CCL19	Q99731
	C-C motif chemokine 20	CCL20	P78556
	C-C motif chemokine 23	CCL23	P55773
	C-C motif chemokine 25	CCL25	O15444
	C-C motif chemokine 28	CCL28	Q9NRJ3
	C-X-C motif chemokine 1	CXCL1	P09341
	C-X-C motif chemokine 5	CXCL5	P42830
	C-X-C motif chemokine 6	CXCL6	P80162
	C-X-C motif chemokine 9	CXCL9	Q07325
	C-X-C motif chemokine 10	CXCL10	P02778
	C-X-C motif chemokine 11	CXCL11	O14625
**Fibroblast growth factor**			
	Fibroblast growth factor 21	FGF-21	Q9NSA1
	Fibroblast growth factor 23	FGF-23	Q9GZV9
	Fibroblast growth factor 19	FGF-19	O95750
**Monocytes chemotactic protein**			
	Monocyte chemotactic protein 1	MCP-1	P13500
	Monocyte chemotactic protein 2	MCP-2	P80075
	Monocyte chemotactic protein 3	MCP-3	P80098
	Monocyte chemotactic protein 4	MCP-4	Q99616
**Tumour necrosis factor**			
	TNF-beta	TNFB	P01374
	TNF-related activation-induced cytokine	TRANCE	O14788

	TNF-related apoptosis-inducing ligand	TRAIL	P50591
	Tumour necrosis factor (Ligand) superfamily, member 12	TWEAK	O43508
	Tumour necrosis factor ligand superfamily, member 14	TNFSF14	O43557
	Tumour necrosis factor receptor superfamily member 9	TNFRSF9	Q07011
**Others**			
	Adenosine Deaminase	ADA	P00813
	Artemin	ARTN	Q5T4W7
	Axin-1	AXIN1	O15169
	Beta-nerve growth factor	Beta-NGF	P01138
	Caspase-8	CASP-8	Q14790
	CD40L-receptor	CD40	P25942
	CUB domain-containing protein 1	CDCP1	Q9H5V8
	Cystatin D	CST5	P28325
	Delta and Notch-like epidemial growth factor-related receptor	DNER	Q8NFT8
	Eotaxin	CCL11	P51671
	Eukaryotic translation initiation factor 4E-binding protein 1	4E-BP1	Q13541
	Fms-related tyrosine kinase 3	Flt3L	P49771
	Fractalkine	CX3CL1	P78423
	Glial cell line-derived neurotrophic factor	GDNF	P39905
	Hepatocyte growth factor	HGF	P14210
	Interferon gamma	IFN-gamma	P01579
	Latency-associated peptide transforming growth factor beta-1	LAP TGF-beta-1	P01137
	Leukemia inhibitory factor	LIF	P15018
	Macrophage colony-stimulating factor1	CSF1	P09603
	Natural killer cell receptor 2B4	CD244	Q9BZW8
	Neurotrophin-3	NT-3	P20783
	Oncostatin-M	OSM	P13725
	Osteoprotegerin	OPG	O00300
	Protein S100-A12	EN-RAGE	P80511
	Signaling lymphocytic activation molecule	SLAMF1	Q13291
	SIR2-like protein 2	SIRT2	Q8IXJ6
	STAM-binding protein	STAMBP	O95630
	Stem cell factor	SCF	P21583
	T-cell surface glycoprotein CD6 isoform	CD6	P30203
	T-cell surface glycoprotein CD5	CD5	P06217
	Urokinase-type plasminogen activator	uPA	P00749

Table S1, Continued

Abbreviation	Biological Process
**Interleukins**	
IL-6	neutrophil mediated immunity, T follicular helper cell differentiation, germinal center B cell differentiation, humoral immune response, hepatic immune response, monocyte chemotaxis, positive regulation of apoptotic DNA fragmentation, positive regulation of transcription by RNA polymerase II, positive regulation of miRNA processing, positive regulation of DNA-binding transcription factor activity, negative regulation of primary miRNA processing, positive regulation of translation, positive regulation of tyrosine phosphorylation of STAT protein, positive regulation of peptidyl-serine phosphorylation, positive regulation of interleukin-1 beta production, positive regulation of interleukin-21 production, positive regulation of tumor necrosis factor production, positive regulation of T-helper 2 cell cytokine production, positive regulation of interleukin-17 production, positive regulation of interleukin-6 production, positive regulation of chemokine production, positive regulation of interleukin-8 production, positive regulation of cytokine production involved in inflammatory response, positive regulation of interleukin-10 production, positive regulation of vascular endothelial growth factor production, positive regulation of immunoglobulin production, negative regulation of chemokine production, negative regulation of collagen biosynthetic process, positive regulation of smooth muscle cell proliferation, positive regulation of glial cell proliferation, positive regulation of T cell proliferation, positive regulation of osteoblast differentiation, positive regulation of epithelial to mesenchymal transition, positive regulation of MAPK cascade, positive regulation of receptor signaling pathway via JAK-STAT, positive regulation of type B pancreatic cell apoptotic process, positive regulation of B cell activation, positive regulation of leukocyte adhesion to vascular endothelial cell, positive regulation of platelet aggregation, positive regulation of leukocyte chemotaxis, positive regulation of extracellular matrix disassembly, negative regulation of cell population proliferation, negative regulation of interleukin-1-mediated signaling pathway, regulation of insulin secretion, regulation of glucagon secretion, negative regulation of fat cell differentiation, negative regulation of neurogenesis, negative regulation of apoptotic process, regulation of astrocyte activation, regulation of microglial cell activation, interleukin-6-mediated signaling pathway, positive regulation of acute inflammatory response, positive regulation of neuroinflammatory response, regulation of angiogenesis, negative regulation of bone resorption, negative regulation of lipid storage, glucagon secretion, cellular response to hydrogen peroxide, acute-phase response, defense response to virus, defense response to Gram-negative bacterium, defense response to Gram-positive bacterium, cellular response to lipopolysaccharide, response to peptidoglycan, response to glucocorticoid, cellular response to virus, response to activity, T-helper 17 cell lineage commitment, hepatocyte proliferation, platelet activation, neutrophil apoptotic process, neuron projection development, endocrine pancreas development, liver regeneration, neuron cellular homeostasis, glucose homeostasis, maintenance of blood-brain barrier, vascular endothelial growth factor production
IL-7	regulation of peptidyl-tyrosine phosphorylation, positive regulation of T cell differentiation, positive regulation of B cell differentiation, positive regulation of B cell proliferation, positive regulation of cytokine-mediated signaling pathway, extrinsic apoptotic signaling pathway, cytokine-mediated signaling pathway, negative regulation of extrinsic apoptotic signaling pathway in absence of ligand, positive regulation of chemokine production, positive regulation of organ growth, cell-cell signaling, negative regulation of catalytic activity, animal organ morphogenesis, T cell lineage commitment,

	B cell proliferation, bone resorption, homeostasis of number of cells within a tissue, organ growth, humoral immune response
IL-8	neutrophil chemotaxis, calcium-mediated signaling, G protein-coupled receptor signaling pathway, chemokine-mediated signaling pathway, response to endoplasmic reticulum stress, cellular response to tumor necrosis factor, cellular response to interleukin-1, cellular response to lipopolysaccharide, neutrophil activation, killing of cells of another organism, antimicrobial humoral immune response mediated by antimicrobial peptide, cellular response to fibroblast growth factor stimulus, inflammatory response, negative regulation of cell adhesion molecule production, negative regulation of G protein-coupled receptor signaling pathway, negative regulation of cell population proliferation, positive regulation of cellular biosynthetic process, regulation of single stranded viral RNA replication via double stranded DNA intermediate, positive regulation of neutrophil chemotaxis, regulation of cell adhesion, negative regulation of gene expression, positive regulation of gene expression, induction of positive chemotaxis, positive regulation of angiogenesis, regulation of entry of bacterium into host cell, receptor internalization, embryonic digestive tract development, angiogenesis
IL-10	cytoplasmic sequestering of NF-kappaB, positive regulation of DNA-binding transcription factor activity, positive regulation of transcription by RNA polymerase II, positive regulation of miRNA transcription, negative regulation of nitric oxide biosynthetic process, regulation of isotype switching, negative regulation of autophagy, negative regulation of membrane protein ectodomain proteolysis, positive regulation of cell cycle, positive regulation of macrophage activation, positive regulation of plasma cell differentiation, positive regulation of heterotypic cell-cell adhesion, positive regulation of endothelial cell proliferation, positive regulation of vascular associated smooth muscle cell proliferation, positive regulation of receptor signaling pathway via JAK-STAT, positive regulation of B cell apoptotic process, negative regulation of hydrogen peroxide-induced neuron death, negative regulation of neuron apoptotic process, negative regulation of mitotic cell cycle, negative regulation of B cell proliferation, negative regulation of T cell proliferation, negative regulation of vascular associated smooth muscle cell proliferation, negative regulation of myeloid dendritic cell activation, negative regulation of cytokine activity, negative regulation of heterotypic cell-cell adhesion, positive regulation of signaling receptor activity, regulation of synapse organization, positive regulation of MHC class II biosynthetic process, negative regulation of MHC class II biosynthetic process, negative regulation of interleukin-18 production, negative regulation of tumor necrosis factor production, negative regulation of interleukin-1 production, negative regulation of interleukin-12 production, negative regulation of cytokine production involved in immune response, negative regulation of interleukin-8 production, negative regulation of type II interferon production, negative regulation of interleukin-6 production, negative regulation of interferon-alpha production, negative regulation of chemokine (C-C motif) ligand 5 production, positive regulation of cytokine production, positive regulation of immunoglobulin production, positive regulation of sprouting angiogenesis, negative regulation of chronic inflammatory response to antigenic stimulus, negative regulation of sensory perception of pain, regulation of response to wounding, cellular response to estradiol stimulus, cellular response to lipopolysaccharide, response to glucocorticoid, response to insulin, response to carbon monoxide, response to xenobiotic stimulus, defense response to protozoan, defense response to bacterium, response to activity, cellular response to hepatocyte growth factor stimulus, chronic inflammatory response to antigenic stimulus, type 2 immune response, response to inactivity, B cell differentiation, B cell proliferation, hemopoiesis, endothelial cell apoptotic process, leukocyte chemotaxis, branching involved in labyrinthine layer morphogenesis, liver regeneration

IL-10RB	immune response, cellular response to virus, defense response to virus, inflammatory response, type III interferon-mediated signaling pathway, positive regulation of receptor signaling pathway via JAK-STAT, positive regulation of cellular respiration
IL-12B	negative regulation of smooth muscle cell proliferation, negative regulation of blood vessel endothelial cell proliferation involved in sprouting angiogenesis, negative regulation of protein secretion, negative regulation of vascular endothelial growth factor signaling pathway, positive regulation of natural killer cell proliferation, positive regulation of activated T cell proliferation, positive regulation of NK T cell proliferation, positive regulation of T-helper 17 cell lineage commitment, positive regulation of memory T cell differentiation, positive regulation of NIK/NF-kappaB signaling, positive regulation of receptor signaling pathway via JAK-STAT, positive regulation of activation of Janus kinase activity, positive regulation of tyrosine phosphorylation of STAT protein, positive regulation of osteoclast differentiation, positive regulation of smooth muscle cell apoptotic process, positive regulation of T cell mediated cytotoxicity, positive regulation of natural killer cell mediated cytotoxicity directed against tumor cell target, cytokine-mediated signaling pathway, negative regulation of interleukin-10 production, negative regulation of interleukin-17 production, negative regulation of inflammatory response to antigenic stimulus, positive regulation of T-helper 1 type immune response, positive regulation of tumor necrosis factor production, positive regulation of interleukin-12 production, positive regulation of type II interferon production, positive regulation of interleukin-17 production, positive regulation of granulocyte macrophage colony-stimulating factor production, positive regulation of interleukin-10 production, positive regulation of tissue remodeling, positive regulation of inflammatory response, positive regulation of defense response to virus by host, T cell proliferation, T-helper cell differentiation, natural killer cell activation involved in immune response, sensory perception of pain, cell migration, cellular response to type II interferon, cellular response to lipopolysaccharide, T-helper 1 type immune response, response to UV-B, defense response to virus, defense response to Gram-negative bacterium, defense response to protozoan, sexual reproduction
IL-18	positive regulation of natural killer cell proliferation, positive regulation of activated T cell proliferation, positive regulation of NK T cell proliferation, positive regulation of T-helper 2 cell differentiation, positive regulation of T-helper 1 cell cytokine production, positive regulation of type II interferon production, positive regulation of interleukin-17 production, positive regulation of chemokine production, positive regulation of interleukin-13 production, positive regulation of granulocyte macrophage colony-stimulating factor production, positive regulation of tissue remodeling, positive regulation of cold-induced thermogenesis, positive regulation of smooth muscle cell proliferation, positive regulation of transcription by RNA polymerase II, positive regulation of tyrosine phosphorylation of STAT protein, activation of protein kinase B activity, positive regulation of macrophage derived foam cell differentiation, positive regulation of NIK/NF-kappaB signaling, positive regulation of protein kinase B signaling, positive regulation of phosphatidylinositol 3-kinase signaling, MAPK cascade, NIK/NF-kappaB signaling, lipopolysaccharide-mediated signaling pathway, interleukin-18-mediated signaling pathway, positive regulation of NF-kappaB transcription factor activity, negative regulation of myoblast differentiation, positive regulation of neuroinflammatory response, cell-cell signaling, natural killer cell activation, neutrophil activation, cell population proliferation, natural killer cell mediated cytotoxicity, cellular response to organic cyclic compound, establishment of skin barrier, angiogenesis, sleep, T-helper 1 type immune response, type 2 immune response, triglyceride homeostasis, cholesterol homeostasis, defense response to Gram-positive bacterium, inflammatory response

IL-18R1	immune response, inflammatory response, natural killer cell activation, T-helper 1 cell differentiation, NIK/NF-kappaB signaling, interleukin-18-mediated signaling pathway, positive regulation of NIK/NF-kappaB signaling, positive regulation of NF-kappaB transcription factor activity, positive regulation of T-helper 1 cell cytokine production, positive regulation of type II interferon production, negative regulation of cold-induced thermogenesis
**Chemokines**	
CCL3	protein kinase B signaling, MAPK cascade, calcium-mediated signaling, lipopolysaccharide-mediated signaling pathway, chemokine-mediated signaling pathway, G protein-coupled receptor signaling pathway, positive regulation of natural killer cell chemotaxis, positive regulation of microglial cell migration, positive regulation of microglial cell activation, positive regulation of neuron apoptotic process, positive regulation of ERK1 and ERK2 cascade, positive regulation of protein kinase B signaling, positive regulation of calcium-mediated signaling, negative regulation of osteoclast differentiation, release of sequestered calcium ion into cytosol by sarcoplasmic reticulum, positive regulation of interleukin-1 beta production, positive regulation of tumor necrosis factor production, negative regulation of gene expression, negative regulation of bone mineralization, regulation of behavior, regulation of sensory perception of pain, positive regulation of calcium ion import, regulation of cell shape, cell-cell signaling, positive regulation of GTPase activity, neutrophil chemotaxis, eosinophil chemotaxis, monocyte chemotaxis, macrophage chemotaxis, T cell chemotaxis, astrocyte cell migration, eosinophil degranulation, cytoskeleton organization, cellular response to tumor necrosis factor, cellular response to interleukin-1, cellular response to type II interferon, cellular response to organic cyclic compound, osteoblast differentiation, intracellular calcium ion homeostasis, response to cholesterol, response to toxic substance, inflammatory response, negative regulation by host of viral transcription
CCL4	establishment or maintenance of cell polarity, neutrophil chemotaxis, eosinophil chemotaxis, monocyte chemotaxis, lymphocyte chemotaxis, G protein-coupled receptor signaling pathway, chemokine-mediated signaling pathway, cell adhesion, cellular response to tumor necrosis factor, cellular response to interleukin-1, cellular response to type II interferon, cell-cell signaling, immune response, positive regulation of natural killer cell chemotaxis, positive regulation of ERK1 and ERK2 cascade, positive regulation of calcium-mediated signaling, positive regulation of calcium ion transport, positive regulation of GTPase activity, response to toxic substance, inflammatory response, response to virus, negative regulation by host of viral transcription

CCL19	positive regulation of interleukin-1 beta production, positive regulation of tumor necrosis factor production, positive regulation of interleukin-12 production, positive regulation of glycoprotein biosynthetic process, positive regulation of JUN kinase activity, positive regulation of phosphatidylinositol 3-kinase activity, positive regulation of T cell proliferation, positive regulation of T-helper 1 cell differentiation, T cell costimulation, positive regulation of dendritic cell dendrite assembly, positive regulation of receptor-mediated endocytosis, positive regulation of NIK/NF-kappaB signaling, positive regulation of I-kappaB kinase/NF-kappaB signaling, positive regulation of JNK cascade, positive regulation of ERK1 and ERK2 cascade, positive regulation of protein kinase B signaling, positive regulation of neutrophil chemotaxis, positive regulation of dendritic cell antigen processing and presentation, release of sequestered calcium ion into cytosol, negative regulation of dendritic cell apoptotic process, G protein-coupled receptor signaling pathway, chemokine-mediated signaling pathway, positive regulation of GTPase activity, neutrophil chemotaxis, myeloid dendritic cell chemotaxis, monocyte chemotaxis, lymphocyte chemotaxis, immunological synapse formation, establishment of T cell polarity, cellular response to tumor necrosis factor, cellular response to interleukin-1, cellular response to type II interferon, cell maturation, mature conventional dendritic cell differentiation, cell communication, killing of cells of another organism, antimicrobial humoral immune response mediated by antimicrobial peptide, inflammatory response, cellular response to virus, response to prostaglandin E, response to nitric oxide, intracellular calcium ion homeostasis
CCL20	neutrophil chemotaxis, monocyte chemotaxis, lymphocyte chemotaxis, thymocyte migration, G protein-coupled receptor signaling pathway, chemokine-mediated signaling pathway, calcium-mediated signaling using intracellular calcium source, cellular response to tumor necrosis factor, cellular response to interleukin-1, cellular response to type II interferon, cell-cell signaling, killing of cells of another organism, antimicrobial humoral immune response mediated by antimicrobial peptide, defense response to bacterium, inflammatory response, positive regulation of ERK1 and ERK2 cascade, positive regulation of T cell migration, positive regulation of GTPase activity
CCL23	neutrophil chemotaxis, monocyte chemotaxis, lymphocyte chemotaxis, G protein-coupled receptor signaling pathway, chemokine-mediated signaling pathway, cellular response to tumor necrosis factor, cellular response to interleukin-1, cellular response to type II interferon, cell-cell signaling, immune response, inflammatory response, positive regulation of ERK1 and ERK2 cascade, negative regulation of cell population proliferation, positive regulation of GTPase activity, negative regulation of C-C chemokine binding, intracellular calcium ion homeostasis
CCL25	neutrophil chemotaxis, monocyte chemotaxis, lymphocyte chemotaxis, chemokine-mediated signaling pathway, G protein-coupled receptor signaling pathway, cellular response to tumor necrosis factor, cellular response to interleukin-1, cellular response to type II interferon, killing of cells of another organism, antimicrobial humoral immune response mediated by antimicrobial peptide, inflammatory response, positive regulation of ERK1 and ERK2 cascade, positive regulation of cell-matrix adhesion, negative regulation of leukocyte tethering or rolling, positive regulation of GTPase activity
CCL28	antimicrobial humoral immune response mediated by antimicrobial peptide, response to nutrient, cytolysis in another organism, cell chemotaxis, positive regulation of cytosolic calcium ion concentration, negative regulation of leukocyte tethering or rolling, positive regulation of cell-matrix adhesion

CXCL1	neutrophil chemotaxis, intracellular signal transduction, G protein-coupled receptor signaling pathway, chemokine-mediated signaling pathway, actin cytoskeleton organization, killing of cells of another organism, cellular response to lipopolysaccharide, antimicrobial humoral immune response mediated by antimicrobial peptide, inflammatory response, negative regulation of cell population proliferation, nervous system development
CXCL5	neutrophil chemotaxis, chemokine-mediated signaling pathway, cell-cell signaling, cellular response to lipopolysaccharide, antimicrobial humoral immune response mediated by antimicrobial peptide, inflammatory response, positive regulation of cell population proliferation
CXCL6	neutrophil chemotaxis, chemokine-mediated signaling pathway, cell-cell signaling, neutrophil activation, cellular response to lipopolysaccharide, antimicrobial humoral immune response mediated by antimicrobial peptide, leukocyte homeostasis, defense response to bacterium, inflammatory response, regulation of neutrophil mediated killing of gram-negative bacterium, regulation of chemokine production
CXCL9	neutrophil chemotaxis, adenylate cyclase-activating G protein-coupled receptor signaling pathway, chemokine-mediated signaling pathway, cell-cell signaling, killing of cells of another organism, cellular response to lipopolysaccharide, antimicrobial humoral immune response mediated by antimicrobial peptide, defense response to virus, inflammatory response, cellular defense response, positive regulation of release of sequestered calcium ion into cytosol, positive regulation of myoblast differentiation, positive regulation of myoblast fusion, regulation of cell population proliferation
CXCL10	neutrophil chemotaxis, T cell chemotaxis, chemokine-mediated signaling pathway, adenylate cyclase-activating G protein-coupled receptor signaling pathway, cellular response to heat, cellular response to interleukin-17, cellular response to lipopolysaccharide, cell-cell signaling, endothelial cell activation, killing of cells of another organism, antimicrobial humoral immune response mediated by antimicrobial peptide, antiviral innate immune response, positive regulation of transcription by RNA polymerase II, positive regulation of monocyte chemotaxis, positive regulation of T cell migration, positive regulation of cell population proliferation, positive regulation of release of sequestered calcium ion into cytosol, regulation of T cell chemotaxis, regulation of apoptotic process, negative regulation of myoblast differentiation, negative regulation of myoblast fusion, negative regulation of angiogenesis, regulation of endothelial tube morphogenesis, inflammatory response, response to cold, cellular response to virus, response to auditory stimulus, response to vitamin D, response to gamma radiation, blood circulation, muscle organ development
CXCL11	neutrophil chemotaxis, T cell chemotaxis, chemokine-mediated signaling pathway, adenylate cyclase-activating G protein-coupled receptor signaling pathway, cell-cell signaling, killing of cells of another organism, cellular response to lipopolysaccharide, antimicrobial humoral immune response mediated by antimicrobial peptide, inflammatory response, positive regulation of release of sequestered calcium ion into cytosol, regulation of cell population proliferation
**Fibroblast growth factor**	

FGF-21	endoplasmic reticulum unfolded protein response, cellular response to low-density lipoprotein particle stimulus, cellular response to glucagon stimulus, cellular response to glucose stimulus, cellular response to xenobiotic stimulus, response to activity, response to methionine, response to nutrient levels, positive regulation of MAPKKK cascade by fibroblast growth factor receptor signaling pathway, endothelial cell apoptotic process, cell-cell signaling, cell differentiation, regulation of cell migration, positive regulation of glucose import, positive regulation of cell population proliferation, positive regulation of triglyceride catabolic process, positive regulation of protein phosphorylation, positive regulation of ERK1 and ERK2 cascade, negative regulation of endothelial cell apoptotic process, negative regulation of neuron death, positive regulation of cold-induced thermogenesis, positive regulation of gene expression, regulation of low-density lipoprotein particle clearance, positive regulation of low-density lipoprotein receptor activity, animal organ morphogenesis
FGF-23	regulation of cell migration, positive regulation of MAPKKK cascade by fibroblast growth factor receptor signaling pathway, ERK1 and ERK2 cascade, positive regulation of cell population proliferation, positive regulation of ERK1 and ERK2 cascade, positive regulation of DNA-templated transcription, positive regulation of protein phosphorylation, negative regulation of osteoblast differentiation, negative regulation of hormone secretion, regulation of phosphate transport, positive regulation of gene expression, negative regulation of bone mineralization, positive regulation of vitamin D 24-hydroxylase activity, cellular response to parathyroid hormone stimulus, cellular response to leptin stimulus, cellular response to vitamin D, cellular response to interleukin-6, vitamin D catabolic process, phosphate-containing compound metabolic process, cell differentiation, response to sodium phosphate, response to magnesium ion, animal organ morphogenesis, calcium ion homeostasis, intracellular phosphate ion homeostasis
FGF-19	regulation of cell migration, fibroblast growth factor receptor signaling pathway, positive regulation of glucose import, positive regulation of cell population proliferation, positive regulation of JNK cascade, positive regulation of ERK1 and ERK2 cascade, positive regulation of protein phosphorylation, negative regulation of bile acid biosynthetic process, positive regulation of gene expression, negative regulation of gene expression, neural crest cell migration, cell differentiation, animal organ morphogenesis, nervous system development, heart development, response to ethanol, response to organic cyclic compound, response to bacterium, bile acid and bile salt transport
**Monocytes chemotactic protein**	

MCP-1	protein kinase B signaling, MAPK cascade, lipopolysaccharide-mediated signaling pathway, chemokine-mediated signaling pathway, receptor signaling pathway via JAK-STAT, G protein-coupled receptor signaling pathway, coupled to cyclic nucleotide second messenger, negative regulation of natural killer cell chemotaxis, negative regulation of vascular endothelial cell proliferation, negative regulation of G1/S transition of mitotic cell cycle, negative regulation of glial cell apoptotic process, negative regulation of neuron apoptotic process, positive regulation of endothelial cell apoptotic process, positive regulation of NMDA glutamate receptor activity, positive regulation of synaptic transmission, glutamatergic, positive regulation of ERK1 and ERK2 cascade, positive regulation of T cell activation, positive regulation of apoptotic cell clearance, positive regulation of calcium ion import, positive regulation of nitric-oxide synthase biosynthetic process, regulation of cell shape, positive regulation of GTPase activity, neutrophil chemotaxis, eosinophil chemotaxis, monocyte chemotaxis, macrophage chemotaxis, lymphocyte chemotaxis, helper T cell extravasation, astrocyte cell migration, protein phosphorylation, cell adhesion, cytoskeleton organization, cellular response to tumor necrosis factor, cellular response to interleukin-1, cellular response to type II interferon, cellular response to organic cyclic compound, cellular response to lipopolysaccharide, humoral immune response, cellular response to fibroblast growth factor stimulus, inflammatory response, response to bacterium, cellular homeostasis, animal organ morphogenesis, angiogenesis, sensory perception of pain, viral genome replication
MCP-2	neutrophil chemotaxis, eosinophil chemotaxis, monocyte chemotaxis, lymphocyte chemotaxis, G protein-coupled receptor signaling pathway, chemokine-mediated signaling pathway, exocytosis, cellular response to tumor necrosis factor, cellular response to interleukin-1, cellular response to type II interferon, cell-cell signaling, killing of cells of another organism, antimicrobial humoral immune response mediated by antimicrobial peptide, inflammatory response, response to virus, negative regulation by host of viral genome replication, positive regulation of ERK1 and ERK2 cascade, positive regulation of leukocyte migration, negative regulation of leukocyte proliferation, positive regulation of GTPase activity, intracellular calcium ion homeostasis, calcium ion transport
MCP-3	neutrophil chemotaxis, eosinophil chemotaxis, monocyte chemotaxis, lymphocyte chemotaxis, G protein-coupled receptor signaling pathway, chemokine-mediated signaling pathway, cytoskeleton organization, cellular response to tumor necrosis factor, cellular response to interleukin-1, cellular response to type II interferon, cellular response to ethanol, cell-cell signaling, positive regulation of natural killer cell chemotaxis, positive regulation of ERK1 and ERK2 cascade, regulation of cell shape, positive regulation of GTPase activity, intracellular calcium ion homeostasis, inflammatory response, response to gamma radiation
MCP-4	neutrophil chemotaxis, eosinophil chemotaxis, monocyte chemotaxis, lymphocyte chemotaxis, G protein-coupled receptor signaling pathway, chemokine-mediated signaling pathway, cytoskeleton organization, cellular response to tumor necrosis factor, cellular response to interleukin-1, cellular response to type II interferon, cell-cell signaling, killing of cells of another organism, antimicrobial humoral immune response mediated by antimicrobial peptide, inflammatory response, positive regulation of ERK1 and ERK2 cascade, regulation of cell shape, positive regulation of GTPase activity, intracellular calcium ion homeostasis
**Tumour necrosis factor**	

TNFB	response to hypoxia, defense response to Gram-positive bacterium, response to nutrient, response to lipopolysaccharide, response to xenobiotic stimulus, humoral immune response, apoptotic process, signal transduction, cell-cell signaling, positive regulation of glial cell proliferation, positive regulation of apoptotic process, negative regulation of fibroblast proliferation, positive regulation of type II interferon production, positive regulation of humoral immune response mediated by circulating immunoglobulin, positive regulation of chronic inflammatory response to antigenic stimulus, lymph node development
TRANCE	protein kinase B signaling, JNK cascade, ERK1 and ERK2 cascade, calcium-mediated signaling, I-kappaB kinase/NF-kappaB signaling, positive regulation of ERK1 and ERK2 cascade via TNFSF11-mediated signaling, positive regulation of transcription by RNA polymerase II, negative regulation of transcription by RNA polymerase II, positive regulation of NF-kappaB transcription factor activity, positive regulation of MAP kinase activity, positive regulation of I-kappaB kinase/NF-kappaB signaling, positive regulation of JNK cascade, positive regulation of protein kinase B signaling, positive regulation of corticotropin-releasing hormone secretion, positive regulation of osteoclast development, positive regulation of fever generation by positive regulation of prostaglandin secretion, positive regulation of T cell activation, positive regulation of homotypic cell-cell adhesion, positive regulation of gene expression, positive regulation of bone resorption, paracrine signaling, regulation of actin binding, osteoclast development, osteoclast differentiation, cellular response to leukemia inhibitory factor, mammary gland epithelial cell proliferation, osteoclast proliferation, monocyte chemotaxis, immune response, tooth eruption, mammary gland alveolus development, ossification, bone resorption, calcium ion homeostasis
TRAIL	apoptotic process, signal transduction, cell-cell signaling, positive regulation of extrinsic apoptotic signaling pathway, positive regulation of I-kappaB kinase/NF-kappaB signaling, positive regulation of release of cytochrome c from mitochondria, positive regulation of cysteine-type endopeptidase activity involved in apoptotic process, immune response, response to insulin, male gonad development
TWEAK	apoptotic process, extrinsic apoptotic signaling pathway, cell differentiation, endothelial cell migration, positive regulation of extrinsic apoptotic signaling pathway, positive regulation of endothelial cell proliferation, positive regulation of angiogenesis, positive regulation of protein catabolic process, immune response, angiogenesis
TNFSF14	apoptotic process, T cell proliferation, signal transduction, cellular response to mechanical stimulus, T cell chemotaxis, T cell homeostasis, immune response, T cell costimulation, positive regulation of NIK/NF-kappaB signaling, positive regulation of myoblast differentiation, positive regulation of myoblast fusion, positive regulation of T cell chemotaxis
TNFRSF9	apoptotic process, regulation of immature T cell proliferation in thymus, negative regulation of cell population proliferation
**Others**	

ADA	apoptotic process, pyroptosis, natural killer cell activation, T cell activation, B cell activation, TRAIL-activated apoptotic signaling pathway, cellular response to mechanical stimulus, cellular response to organic cyclic compound, macrophage differentiation, syncytiotrophoblast cell differentiation involved in labyrinthine layer development, execution phase of apoptosis, positive regulation of interleukin-1 beta production, activation of cysteine-type endopeptidase activity involved in apoptotic process, activation of cysteine-type endopeptidase activity, positive regulation of macrophage differentiation, positive regulation of I-kappaB kinase/NF-kappaB signaling, positive regulation of apoptotic process, positive regulation of neuron death, negative regulation of I-kappaB kinase/NF-kappaB signaling, regulation of tumor necrosis factor-mediated signaling pathway, negative regulation of necroptotic process, regulation of innate immune response, self proteolysis, proteolysis involved in protein catabolic process, response to tumor necrosis factor, response to lipopolysaccharide, response to estradiol, response to ethanol, response to antibiotic, response to cobalt ion, response to cold, angiogenesis, heart development
ARTN	lymphocyte migration into lymphoid organs, signal transduction, axon guidance, neuroblast proliferation, induction of positive chemotaxis, peripheral nervous system development, Peyer's patch morphogenesis
AXIN1	apoptotic process, cell development, canonical Wnt signaling pathway, protein-containing complex assembly, genomic imprinting, cytoplasmic microtubule organization, nucleocytoplasmic transport, positive regulation of proteasomal ubiquitin-dependent protein catabolic process, activation of protein kinase activity, positive regulation of JUN kinase activity, positive regulation of peptidyl-threonine phosphorylation, positive regulation of peptidyl-serine phosphorylation, positive regulation of ubiquitin-protein transferase activity, negative regulation of protein metabolic process, negative regulation of transcription elongation by RNA polymerase II, positive regulation of DNA-templated transcription, positive regulation of JNK cascade, positive regulation of transforming growth factor beta receptor signaling pathway, negative regulation of canonical Wnt signaling pathway, negative regulation of fat cell differentiation, proteasome-mediated ubiquitin-dependent protein catabolic process, protein polyubiquitination, axial mesoderm formation, in utero embryonic development, head development, post-anal tail morphogenesis, dorsal/ventral axis specification, sensory perception of sound
Beta-NGF	nerve development, peripheral nervous system development, neuron projection morphogenesis, neuron apoptotic process, nerve growth factor signaling pathway, extrinsic apoptotic signaling pathway via death domain receptors, transmembrane receptor protein tyrosine kinase signaling pathway, modulation of chemical synaptic transmission, positive regulation of Ras protein signal transduction, positive regulation of neuron differentiation, positive regulation of collateral sprouting, positive regulation of peptidyl-serine phosphorylation, negative regulation of cell population proliferation, negative regulation of neuron apoptotic process, activation of cysteine-type endopeptidase activity involved in apoptotic process, positive regulation of gene expression, positive regulation of DNA binding, memory

CASP-8	apoptotic process, pyroptosis, natural killer cell activation, T cell activation, B cell activation, TRAIL-activated apoptotic signaling pathway, cellular response to mechanical stimulus, cellular response to organic cyclic compound, macrophage differentiation, syncytiotrophoblast cell differentiation involved in labyrinthine layer development, execution phase of apoptosis, positive regulation of interleukin-1 beta production, activation of cysteine-type endopeptidase activity involved in apoptotic process, activation of cysteine-type endopeptidase activity, positive regulation of macrophage differentiation, positive regulation of I-kappaB kinase/NF-kappaB signaling, positive regulation of apoptotic process, positive regulation of neuron death, negative regulation of I-kappaB kinase/NF-kappaB signaling, regulation of tumor necrosis factor-mediated signaling pathway, negative regulation of necroptotic process, regulation of innate immune response, self proteolysis, proteolysis involved in protein catabolic process, response to tumor necrosis factor, response to lipopolysaccharide, response to estradiol, response to ethanol, response to antibiotic, response to cobalt ion, response to cold, angiogenesis, heart development
CD40	protein kinase B signaling, immune response-regulating cell surface receptor signaling pathway, CD40 signaling pathway, TRIF-dependent toll-like receptor signaling pathway, positive regulation of transcription by RNA polymerase II, positive regulation of NF-kappaB transcription factor activity, positive regulation of isotype switching to IgG isotypes, positive regulation of tyrosine phosphorylation of STAT protein, positive regulation of MAP kinase activity, positive regulation of blood vessel endothelial cell migration, positive regulation of endothelial cell apoptotic process, positive regulation of I-kappaB kinase/NF-kappaB signaling, positive regulation of protein kinase C signaling, positive regulation of B cell proliferation, positive regulation of interleukin-12 production, positive regulation of angiogenesis, positive regulation of GTPase activity, cellular response to tumor necrosis factor, cellular response to interleukin-1, cellular response to mechanical stimulus, protein-containing complex assembly, B cell proliferation, platelet activation, B cell mediated immunity, intracellular calcium ion homeostasis, response to type II interferon, defense response to virus, defense response to protozoan, inflammatory response
CDCP1	
CST5	negative regulation of cysteine-type endopeptidase activity
DNER	central nervous system development, skeletal muscle fiber development, glial cell differentiation, Notch signaling pathway, synapse assembly, neuron migration, Notch receptor processing, endocytosis
CCL11	neutrophil chemotaxis, eosinophil chemotaxis, mast cell chemotaxis, monocyte chemotaxis, lymphocyte chemotaxis, G protein-coupled receptor signaling pathway, chemokine-mediated signaling pathway, ERK1 and ERK2 cascade, protein phosphorylation, cell adhesion, cytoskeleton organization, positive regulation of actin filament polymerization, actin filament organization, cellular response to tumor necrosis factor, cellular response to interleukin-1, cellular response to type II interferon, killing of cells of another organism, antimicrobial humoral immune response mediated by antimicrobial peptide, response to interleukin-13, response to interleukin-4, chronic inflammatory response, response to virus, response to radiation, positive regulation of endothelial cell proliferation, positive regulation of cell migration, positive regulation of ERK1 and ERK2 cascade, negative regulation of neurogenesis, positive regulation of angiogenesis, regulation of cell shape, positive regulation of GTPase activity, intracellular calcium ion homeostasis, branching involved in mammary gland duct morphogenesis, learning or memory, mammary duct terminal end bud growth
4E-BP1	G1/S transition of mitotic cell cycle, TOR signaling, negative regulation of translational initiation, positive regulation of mitotic cell cycle

Flt3L	embryonic hemopoiesis, signal transduction, positive regulation of cell population proliferation
CX3CL1	negative regulation of microglial cell activation, positive regulation of microglial cell migration, negative regulation of interleukin-1 alpha production, negative regulation of interleukin-1 beta production, negative regulation of tumor necrosis factor production, negative regulation of interleukin-6 production, positive regulation of transforming growth factor beta1 production, positive regulation of neuroblast proliferation, negative regulation of neuron migration, negative regulation of extrinsic apoptotic signaling pathway in absence of ligand, negative regulation of hippocampal neuron apoptotic process, negative regulation of glutamate receptor signaling pathway, negative regulation of cell-substrate adhesion, positive regulation of I-kappaB phosphorylation, positive regulation of transcription by RNA polymerase II, positive regulation of NF-kappaB transcription factor activity, positive regulation of smooth muscle cell proliferation, positive regulation of I-kappaB kinase/NF-kappaB signaling, positive regulation of ERK1 and ERK2 cascade, positive regulation of protein kinase B signaling, positive regulation of calcium-independent cell-cell adhesion, positive regulation of cell-matrix adhesion, positive regulation of neuron projection development, positive regulation of actin filament bundle assembly, positive regulation of release of sequestered calcium ion into cytosol, regulation of lipopolysaccharide-mediated signaling pathway, regulation of synaptic plasticity, G protein-coupled receptor signaling pathway, extrinsic apoptotic signaling pathway in absence of ligand, chemokine-mediated signaling pathway, positive regulation of inflammatory response, autocrine signaling, positive regulation of GTPase activity, neutrophil chemotaxis, eosinophil chemotaxis, monocyte chemotaxis, lymphocyte chemotaxis, leukocyte migration involved in inflammatory response, integrin activation, synapse pruning, cell-cell adhesion, leukocyte adhesive activation, microglial cell activation, cellular response to tumor necrosis factor, cellular response to interleukin-1, cellular response to type II interferon, neuron remodeling, microglial cell proliferation, immune response, positive chemotaxis, inflammatory response, response to ischemia, neuron cellular homeostasis, angiogenesis involved in wound healing
GDNF	sympathetic nervous system development, peripheral nervous system development, enteric nervous system development, nervous system development, postganglionic parasympathetic fiber development, metanephros development, embryonic organ development, dorsal spinal cord development, ureteric bud formation, organ induction, mesenchymal to epithelial transition involved in metanephros morphogenesis, branching involved in ureteric bud morphogenesis, mRNA stabilization, positive regulation of transcription by RNA polymerase II, negative regulation of extrinsic apoptotic signaling pathway in absence of ligand, negative regulation of neuron apoptotic process, positive regulation of cell population proliferation, positive regulation of mesenchymal to epithelial transition involved in metanephros morphogenesis, positive regulation of dopamine secretion, signal transduction, regulation of stem cell differentiation, regulation of semaphorin-plexin signaling pathway, positive regulation of ureteric bud formation, positive regulation of branching involved in ureteric bud morphogenesis, regulation of dopamine uptake involved in synaptic transmission, positive regulation of monooxygenase activity, postsynaptic membrane organization, commissural neuron axon guidance, neural crest cell migration, adult locomotory behavior, peristalsis

HGF	positive regulation of transcription by RNA polymerase II, positive regulation of DNA biosynthetic process, positive regulation of peptidyl-tyrosine phosphorylation, regulation of tau-protein kinase activity, negative regulation of peptidyl-serine phosphorylation, negative regulation of autophagy, positive regulation of neuron projection regeneration, positive regulation of MAPK cascade, positive regulation of phosphatidylinositol 3-kinase signaling, positive regulation of osteoblast differentiation, positive regulation of cell migration, positive regulation of myelination, negative regulation of release of cytochrome c from mitochondria, regulation of p38MAPK cascade, negative regulation of hydrogen peroxide-mediated programmed cell death, negative regulation of extrinsic apoptotic signaling pathway via death domain receptors, negative regulation of cysteine-type endopeptidase activity involved in apoptotic process, hepatocyte growth factor receptor signaling pathway, negative regulation of interleukin-6 production, positive regulation of interleukin-10 production, positive regulation of angiogenesis, regulation of branching involved in salivary gland morphogenesis by mesenchymal-epithelial signaling, negative regulation of inflammatory response, liver development, animal organ regeneration, cell morphogenesis, epithelial to mesenchymal transition, skeletal muscle cell proliferation, myoblast proliferation, epithelial cell proliferation, cell chemotaxis, mitotic cell cycle, cellular response to hepatocyte growth factor stimulus
IFN-gamma	negative regulation of epithelial cell differentiation, positive regulation of neurogenesis, positive regulation of osteoclast differentiation, positive regulation of CD4-positive, CD25-positive, alpha-beta regulatory T cell differentiation involved in immune response, negative regulation of smooth muscle cell proliferation, negative regulation of tau-protein kinase activity, negative regulation of transcription by RNA polymerase II, positive regulation of cell population proliferation, positive regulation of protein serine/threonine kinase activity, positive regulation of tyrosine phosphorylation of STAT protein, positive regulation of peptidyl-serine phosphorylation of STAT protein, positive regulation of fructose 1,6-bisphosphate metabolic process, positive regulation of fructose 1,6-bisphosphate 1-phosphatase activity, positive regulation of vitamin D biosynthetic process, positive regulation of nitric oxide biosynthetic process, positive regulation of glycolytic process, positive regulation of autophagy, positive regulation of membrane protein ectodomain proteolysis, positive regulation of cellular respiration, type II interferon-mediated signaling pathway, extrinsic apoptotic signaling pathway, receptor signaling pathway via JAK-STAT, positive regulation of iron ion import across plasma membrane, positive regulation of NMDA glutamate receptor activity, positive regulation of phagocytosis, positive regulation of protein-containing complex assembly, positive regulation of protein import into nucleus, positive regulation of protein localization to plasma membrane, positive regulation of exosomal secretion, positive regulation of smooth muscle cell apoptotic process, positive regulation of killing of cells of another organism, positive regulation of neuron death, positive regulation of epithelial cell migration, regulation of insulin secretion, positive regulation of tumor necrosis factor (ligand) superfamily member 11 production, positive regulation of tumor necrosis factor production, positive regulation of interleukin-1 beta production, positive regulation of interleukin-12 production, positive regulation of interleukin-23 production, positive regulation of interleukin-6 production, positive regulation of chemokine production, negative regulation of interleukin-17 production, negative regulation of amyloid-beta clearance, positive regulation of MHC class II biosynthetic process, positive regulation of nitric-oxide synthase biosynthetic process, positive regulation of protein deacetylation, positive regulation of amyloid-beta formation, positive regulation of inflammatory response, regulation of growth, regulation of protein ADP-ribosylation, positive regulation of calcidiol 1-monooxygenase activity, positive regulation of core promoter binding, apoptotic process, macrophage activation involved in immune response, microglial cell activation, astrocyte activation, macrophage differentiation, adaptive

	immune response, humoral immune response, cellular response to virus, defense response to virus, neuroinflammatory response
LAP TGF-beta-1	common-partner SMAD protein phosphorylation, pathway-restricted SMAD protein phosphorylation, membrane protein intracellular domain proteolysis, ATP biosynthetic process, receptor catabolic process, hyaluronan catabolic process, protein kinase B signaling, MAPK cascade, SMAD protein signal transduction, BMP signaling pathway, transforming growth factor beta receptor signaling pathway involved in heart development, epidermal growth factor receptor signaling pathway, lipopolysaccharide-mediated signaling pathway, extrinsic apoptotic signaling pathway, macrophage derived foam cell differentiation, chondrocyte differentiation, vasculogenesis, epithelial to mesenchymal transition, hematopoietic progenitor cell differentiation, cell migration, SMAD protein complex assembly, extracellular matrix assembly, cell-cell junction organization, cellular response to organic cyclic compound, protein export from nucleus, negative regulation of cell growth, negative regulation of miRNA transcription, negative regulation of miRNA processing, negative regulation of protein phosphorylation, negative regulation of protein localization to plasma membrane, negative regulation of fat cell differentiation, negative regulation of myoblast differentiation, negative regulation of cell cycle, negative regulation of extracellular matrix disassembly, negative regulation of cell-cell adhesion, negative regulation of epithelial cell proliferation, negative regulation of blood vessel endothelial cell migration, negative regulation of cytolysis, positive regulation of extracellular matrix assembly, positive regulation of protein-containing complex assembly, positive regulation of transcription by RNA polymerase II, positive regulation of miRNA transcription, positive regulation of miRNA processing, positive regulation of isotype switching to IgA isotypes, positive regulation of superoxide anion generation, positive regulation of protein dephosphorylation, positive regulation of phosphatidylinositol 3-kinase activity, positive regulation of MAP kinase activity, positive regulation of peptidyl-tyrosine phosphorylation, positive regulation of peptidyl-threonine phosphorylation, positive regulation of pathway-restricted SMAD protein phosphorylation, positive regulation of peptidyl-serine phosphorylation, positive regulation of blood vessel endothelial cell migration, positive regulation of fibroblast migration, positive regulation of smooth muscle cell proliferation, positive regulation of protein secretion, positive regulation of protein import into nucleus, positive regulation of SMAD protein signal transduction, positive regulation of ERK1 and ERK2 cascade, positive regulation of protein kinase B signaling, positive regulation of receptor signaling pathway via STAT, positive regulation of canonical Wnt signaling pathway, positive regulation of cardiac muscle cell differentiation, positive regulation of microglia differentiation, positive regulation of epithelial to mesenchymal transition, positive regulation of cell division, regulation of transforming growth factor beta receptor signaling pathway, negative regulation of hyaluronan biosynthetic process, negative regulation of macrophage cytokine production, negative regulation of skeletal muscle tissue development, negative regulation of biomineral tissue development, positive regulation of collagen biosynthetic process, positive regulation of interleukin-17 production, positive regulation of vascular endothelial growth factor production, positive regulation of chemotaxis, positive regulation of bone mineralization, regulation of blood vessel remodeling, regulation of striated muscle tissue development, positive regulation of transcription regulatory region DNA binding, positive regulation of NAD+ ADP-ribosyltransferase activity, positive regulation of vascular permeability, ossification involved in bone remodeling, connective tissue replacement involved in inflammatory response wound healing, neural tube closure, lymph node development, embryonic liver development, heart development, neural tube development, salivary gland morphogenesis, ventricular cardiac muscle tissue morphogenesis, aortic valve

	morphogenesis, inflammatory response, response to wounding, response to cholesterol, response to progesterone, response to estradiol, cellular response to transforming growth factor beta stimulus
LIF	positive regulation of transcription by RNA polymerase II, positive regulation of tyrosine phosphorylation of STAT protein, positive regulation of peptidyl-serine phosphorylation of STAT protein, positive regulation of fibroblast proliferation, positive regulation of MAPK cascade, positive regulation of astrocyte differentiation, positive regulation of macrophage differentiation, positive regulation of mesenchymal to epithelial transition involved in metanephros morphogenesis, positive regulation of cell adhesion mediated by integrin, positive regulation of protein localization to nucleus, negative regulation of cell population proliferation, negative regulation of ERK1 and ERK2 cascade, negative regulation of hormone secretion, regulation of metanephric nephron tubule epithelial cell differentiation, negative regulation of meiotic nuclear division, leukemia inhibitory factor signaling pathway, positive regulation of gene expression, regulation of RNA polymerase II regulatory region sequence-specific DNA binding, immune response, lung alveolus development, lung vasculature development, decidualization, neuron development, stem cell differentiation, spongiotrophoblast differentiation, trophoblast giant cell differentiation, macrophage differentiation, muscle organ morphogenesis, lung lobe morphogenesis, somatic stem cell population maintenance, meiotic nuclear division, embryo implantation, fibroblast proliferation, tyrosine phosphorylation of STAT protein, blood vessel remodeling
CSF-1	branching involved in mammary gland duct morphogenesis, developmental process involved in reproduction, homeostasis of number of cells within a tissue, inflammatory response, innate immune response, macrophage colony-stimulating factor signaling pathway, macrophage differentiation, macrophage homeostasis, mammary duct terminal end bud growth, mammary gland fat development, microglial cell proliferation, monocyte activation, monocyte differentiation, monocyte homeostasis, myeloid leukocyte migration, negative regulation of neuron death, neutrophil homeostasis, odontogenesis, ossification, osteoclast differentiation, osteoclast proliferation, positive regulation of cell migration, positive regulation of cell population proliferation, positive regulation of cell-matrix adhesion, positive regulation of gene expression, positive regulation of macrophage chemotaxis, positive regulation of macrophage colony-stimulating factor signaling pathway, positive regulation of macrophage derived foam cell differentiation, positive regulation of macrophage differentiation, positive regulation of macrophage migration, positive regulation of microglial cell migration, positive regulation of monocyte differentiation, positive regulation of mononuclear cell proliferation, positive regulation of multicellular organism growth, positive regulation of odontogenesis of dentin-containing tooth, positive regulation of osteoclast differentiation. positive regulation of protein kinase activity, positive regulation of protein metabolic process, positive regulation of Ras protein signal transduction, Ras protein signal transduction, regulation of macrophage derived foam cell differentiation, regulation of ossification, response to ischemia, transmembrane receptor protein tyrosine kinase signaling pathway
CD244	signal transduction, positive regulation of inositol phosphate biosynthetic process, positive regulation of type II interferon production, positive regulation of interleukin-8 production, positive regulation of granzyme B production, natural killer cell activation involved in immune response, innate immune response, adaptive immune response

NT-3	nerve development, peripheral nervous system development, nervous system development, neuron projection morphogenesis, positive regulation of actin cytoskeleton reorganization, positive regulation of receptor internalization, positive regulation of cell population proliferation, activation of protein kinase B activity, positive regulation of MAP kinase activity, positive regulation of peptidyl-tyrosine phosphorylation, positive regulation of peptidyl-serine phosphorylation, positive regulation of cell migration, nerve growth factor signaling pathway, transmembrane receptor protein tyrosine kinase signaling pathway, negative regulation of neuron apoptotic process, modulation of chemical synaptic transmission, negative regulation of peptidyl-tyrosine phosphorylation, regulation of neuron differentiation, induction of positive chemotaxis, cell-cell signaling, activation of GTPase activity, memory
OSM	positive regulation of transcription by RNA polymerase II, positive regulation of tyrosine phosphorylation of STAT protein, positive regulation of peptidyl-serine phosphorylation, positive regulation of cell population proliferation, positive regulation of MAPK cascade, positive regulation of protein kinase B signaling, positive regulation of phosphatidylinositol 3-kinase signaling, positive regulation of cell division, negative regulation of cell population proliferation, negative regulation of hormone secretion, regulation of hematopoietic stem cell differentiation, oncostatin-M-mediated signaling pathway, positive regulation of interleukin-17 production, positive regulation of acute inflammatory response, regulation of growth, immune response
OPG	apoptotic process, signal transduction, extracellular matrix organization, response to estrogen, response to nutrient, response to arsenic-containing substance, response to xenobiotic stimulus, response to magnesium ion, negative regulation of osteoclast differentiation, negative regulation of bone resorption, negative regulation of odontogenesis of dentin-containing tooth, skeletal system development
EN-RAGE	defense response to fungus, antimicrobial humoral immune response mediated by antimicrobial peptide, innate immune response, defense response to bacterium, inflammatory response, killing of cells of another organism, neutrophil chemotaxis, monocyte chemotaxis, xenobiotic metabolic process, mast cell activation, positive regulation of I-kappaB kinase/NF-kappaB signaling, positive regulation of MAP kinase activity, positive regulation of NF-kappaB transcription factor activity, positive regulation of inflammatory response
SLAMF1	natural killer cell proliferation, natural killer cell differentiation, myeloid dendritic cell activation involved in immune response, cell adhesion, leukocyte chemotaxis involved in inflammatory response, innate immune response, adaptive immune response, negative regulation of CD40 signaling pathway, positive regulation of JNK cascade, positive regulation of ERK1 and ERK2 cascade, positive regulation of activated T cell proliferation, positive regulation of dendritic cell chemotaxis, positive regulation of macrophage chemotaxis, regulation of vesicle fusion, negative regulation of T cell cytokine production, negative regulation of tumor necrosis factor production, negative regulation of interleukin-12 production, negative regulation of type II interferon production, negative regulation of interleukin-6 production, positive regulation of T-helper 1 cell cytokine production, positive regulation of type II interferon production, regulation of catalytic activity, phagocytosis

SIRT2	cellular response to epinephrine stimulus, cellular response to molecule of bacterial origin, cellular response to oxidative stress, cellular response to hypoxia, negative regulation of transcription from RNA polymerase II promoter in response to hypoxia, cellular response to caloric restriction, cellular response to hepatocyte growth factor stimulus, innate immune response, response to redox state, protein kinase B signaling, phosphatidylinositol 3-kinase signaling, hepatocyte growth factor receptor signaling pathway, autophagy, cellular lipid catabolic process, rDNA heterochromatin formation, subtelomeric heterochromatin formation, NLRP3 inflammasome complex assembly, mitotic nuclear membrane reassembly, cell division, myelination in peripheral nervous system, meiotic cell cycle, positive regulation of transcription by RNA polymerase II, negative regulation of peptidyl-threonine phosphorylation, negative regulation of autophagy, negative regulation of reactive oxygen species metabolic process, positive regulation of oocyte maturation, positive regulation of attachment of spindle microtubules to kinetochore, positive regulation of meiotic nuclear division, positive regulation of execution phase of apoptosis, positive regulation of cell division, negative regulation of NLRP3 inflammasome complex assembly, negative regulation of oligodendrocyte progenitor proliferation, negative regulation of fat cell differentiation, regulation of exit from mitosis, regulation of myelination, positive regulation of proteasomal ubiquitin-dependent protein catabolic process involved in cellular response to hypoxia, negative regulation of protein catabolic process, negative regulation of striated muscle tissue development, negative regulation of defense response to bacterium, positive regulation of DNA binding, proteasome-mediated ubiquitin-dependent protein catabolic process, peptidyl-lysine deacetylation, tubulin deacetylation, histone H4 deacetylation, histone H3 deacetylation, substantia nigra development
STAMBP	positive regulation of cell population proliferation, negative regulation of Ras protein signal transduction, negative regulation of phosphatidylinositol 3-kinase signaling, negative regulation of hippocampal neuron apoptotic process, receptor signaling pathway via JAK-STAT, hippocampal neuron apoptotic process, mitotic cytokinesis, protein K63-linked deubiquitination
SCF	peptidyl-tyrosine phosphorylation, T cell proliferation, mast cell proliferation, embryonic hemopoiesis, ectopic germ cell programmed cell death, myeloid leukocyte differentiation, hematopoietic progenitor cell differentiation, mast cell apoptotic process, cell adhesion, Ras protein signal transduction, extrinsic apoptotic signaling pathway in absence of ligand, neural crest cell migration, melanocyte migration, mast cell migration, male gonad development, ovarian follicle development, positive regulation of melanocyte differentiation, positive regulation of myeloid leukocyte differentiation, positive regulation of hematopoietic progenitor cell differentiation, positive regulation of mast cell proliferation, positive regulation of T cell proliferation, positive regulation of hematopoietic stem cell proliferation, positive regulation of peptidyl-tyrosine phosphorylation, positive regulation of MAP kinase activity, positive regulation of Ras protein signal transduction, positive regulation of leukocyte migration, negative regulation of mast cell apoptotic process
CD6	immunological synapse formation, lipopolysaccharide-mediated signaling pathway, heterophilic cell-cell adhesion via plasma membrane cell adhesion molecules, positive regulation of T cell proliferation, positive regulation of protein phosphorylation, positive regulation of cytokine production involved in inflammatory response, response to lipopolysaccharide, innate immune response, acute inflammatory response to antigenic stimulus, adaptive immune response
CD5	apoptotic signaling pathway, T cell costimulation, cell recognition

uPA	response to hypoxia, negative regulation of fibrinolysis, fibrinolysis, regulation of signaling receptor activity, negative regulation of plasminogen activation, positive regulation of cell migration, urokinase plasminogen activator signaling pathway, regulation of smooth muscle cell-matrix adhesion, regulation of cell adhesion mediated by integrin, regulation of smooth muscle cell migration, regulation of cell population proliferation, blood coagulation, smooth muscle cell migration, plasminogen activation, chemotaxis
VEGF-A	positive regulation of peptidyl-tyrosine autophosphorylation, activation of protein kinase activity, positive regulation of MAP kinase activity, positive regulation of peptidyl-serine phosphorylation, positive regulation of transcription from RNA polymerase II promoter in response to hypoxia, positive regulation of DNA biosynthetic process, negative regulation of transcription by RNA polymerase II, positive regulation of CREB transcription factor activity, positive regulation of protein kinase D signaling, positive regulation of p38MAPK cascade, positive regulation of ERK1 and ERK2 cascade, positive regulation of protein kinase B signaling, positive regulation of phosphatidylinositol 3-kinase signaling, positive regulation of protein kinase C signaling, positive regulation of focal adhesion assembly, positive regulation of cell migration involved in sprouting angiogenesis, positive regulation of endothelial cell chemotaxis by VEGF-activated vascular endothelial growth factor receptor signaling pathway, positive regulation of mast cell chemotaxis, positive regulation of trophoblast cell migration, positive regulation of blood vessel endothelial cell proliferation involved in sprouting angiogenesis, positive regulation of cell proliferation by VEGF-activated platelet derived growth factor receptor signaling pathway, positive regulation of neuroblast proliferation, positive regulation of receptor internalization, positive regulation of retinal ganglion cell axon guidance, positive regulation of axon extension involved in axon guidance, positive regulation of protein-containing complex assembly, positive regulation of protein localization to early endosome, positive regulation of cell division, regulation of nitric oxide mediated signal transduction, negative regulation of cell-cell adhesion mediated by cadherin, vascular endothelial growth factor receptor-2 signaling pathway, VEGF-activated neuropilin signaling pathway, negative regulation of adherens junction organization, negative regulation of establishment of endothelial barrier, negative regulation of cysteine-type endopeptidase activity involved in apoptotic process, positive regulation of histone deacetylase activity, positive regulation of gene expression, negative regulation of gene expression, positive regulation of cold-induced thermogenesis, induction of positive chemotaxis, positive regulation of epithelial tube formation, positive regulation of sprouting angiogenesis, positive regulation of branching involved in ureteric bud morphogenesis, regulation of cell shape, negative regulation of blood-brain barrier permeability, positive regulation of vascular permeability, lactation, cellular response to hypoxia, cellular stress response to acid chemical, cellular response to vascular endothelial growth factor stimulus, camera-type eye morphogenesis, heart morphogenesis, coronary artery morphogenesis, coronary vein morphogenesis, branching involved in blood vessel morphogenesis, outflow tract morphogenesis, lymph vessel morphogenesis, monocyte differentiation, macrophage differentiation, primitive erythrocyte differentiation, epithelial cell differentiation, vasculogenesis, dopaminergic neuron differentiation, cell maturation, cardiac muscle cell development, cardiac vascular smooth muscle cell development, eye photoreceptor cell development, post-embryonic camera-type eye development, kidney development, lung development, ovarian follicle development, nervous system development, mesoderm development, in utero embryonic development, mammary gland alveolus development, vascular wound healing, sprouting angiogenesis, tube formation, endothelial cell chemotaxis, basophil chemotaxis, cell migration involved in sprouting angiogenesis, motor neuron migration, commissural neuron axon guidance, positive chemotaxis, surfactant homeostasis

Table S2: Odds ratios and 95% confidence intervals for poor outcome 3-months after ischemic stroke per doubling of protein levels as compared to good outcome.

**Protein Name**	**FDR significant All_Univariate**	**All cases**					
		**All_Univariate_OR**	**All_Uni_p.value**	**All_model1_OR**	**All_model1_p.value**	**All_Model2_OR**	**All_model2_p.value**
IL-8		1.49 (1.16-1.92)	1.72E-03	1.55 (1.19-2.02)	1.20E-03	1.34 (0.95-1.87)	8.84E-02
VEGF-A		2.05 (1.32-3.19)	1.40E-03	2.11 (1.33-3.36)	1.56E-03	1.78 (0.94-3.29)	7.08E-02
MCP-3	x	**2.09 (1.56-2.83)**	**1.16E-06**	**2.16 (1.58-2.99)**	**1.72E-06**	1.58 (1.01-2.44)	3.97E-02
GDNF		2.08 (1.32-3.31)	1.69E-03	1.91 (1.19-3.10)	7.69E-03	1.26 (0.66-2.36)	4.78E-01
CDCP1		1.33 (0.97-1.83)	8.01E-02	1.29 (0.91-1.82)	1.48E-01	0.91 (0.56-1.46)	6.98E-01
CD244		0.88 (0.51-1.49)	6.29E-01	0.94 (0.53-1.65)	8.27E-01	1.28 (0.60-2.71)	5.22E-01
IL-7		1.34 (1.03-1.74)	2.72E-02	1.38 (1.05-1.82)	2.04E-02	1.27 (0.88-1.84)	2.08E-01
OPG		1.69 (1.03-2.78)	3.76E-02	1.75 (1.03-2.99)	3.94E-02	1.21 (0.60-2.43)	5.99E-01
LAP TGF-beta-1		1.48 (1.02-2.13)	3.84E-02	1.44 (0.97-2.14)	6.93E-02	1.52 (0.88-2.62)	9.64E-02
uPA		0.40 (0.22-0.71)	1.88E-03	0.38 (0.21-0.69)	1.59E-03	0.86 (0.42-1.72)	6.75E-01
IL-6	x	**1.96 (1.63-2.38)**	**5.31E-12**	**1.98 (1.63-2.43)**	**1.25E-11**	1.39 (1.10-1.75)	5.33E-03
MCP-1		1.15 (0.76-1.71)	5.11E-01	1.05 (0.69-1.60)	8.17E-01	1.31 (0.75-2.29)	3.41E-01
CXCL11		1.10 (0.91-1.34)	3.14E-01	1.14 (0.92-1.39)	2.21E-01	1.12 (0.84-1.48)	4.48E-01
AXIN1		1.25 (1.07-1.47)	4.32E-03	1.28 (1.08-1.51)	3.38E-03	1.37 (1.10-1.71)	5.02E-03
TRAIL	x	**0.19 (0.11-0.32)**	**6.53E-10**	**0.18 (0.10-0.30)**	**4.61E-10**	0.37 (0.18-0.74)	5.39E-03
CXCL9		0.96 (0.77-1.19)	7.03E-01	0.96 (0.76-1.20)	7.34E-01	1.13 (0.83-1.52)	4.23E-01
CST5		0.69 (0.44-1.05)	8.57E-02	0.65 (0.41-1.02)	6.32E-02	1.09 (0.59-1.98)	7.90E-01
OSM	x	**1.96 (1.60-2.43)**	**2.70E-10**	**1.96 (1.59-2.44)**	**6.97E-10**	**1.55 (1.18-2.05)**	**2.00E-03**
CXCL1		1.15 (0.96-1.37)	1.22E-01	1.17 (0.98-1.40)	9.20E-02	1.14 (0.89-1.46)	2.97E-01
CCL4		1.42 (1.03-1.96)	2.98E-02	1.44 (1.04-2.00)	2.78E-02	1.25 (0.79-1.96)	3.28E-01
CD6	x	**0.51 (0.34-0.75)**	**7.39E-04**	**0.49 (0.33-0.74)**	**6.59E-04**	0.70 (0.41-1.18)	1.86E-01
SCF	x	**0.51 (0.35-0.73)**	**2.56E-04**	**0.51 (0.35-0.73)**	**3.20E-04**	0.88 (0.53-1.48)	6.22E-01
IL-18		1.07 (0.76-1.50)	7.11E-01	0.94 (0.65-1.35)	7.40E-01	1.07 (0.66-1.73)	7.89E-01
SLAMF1		1.38 (0.96-1.99)	7.94E-02	1.28 (0.88-1.87)	1.98E-01	0.94 (0.55-1.57)	8.08E-01
MCP-4		0.86 (0.62-1.17)	3.50E-01	0.82 (0.59-1.14)	2.43E-01	0.88 (0.56-1.37)	5.80E-01
CCL11		0.66 (0.44-0.97)	3.82E-02	0.56 (0.36-0.85)	8.18E-03	0.92 (0.53-1.58)	7.56E-01
TNFSF14	x	**3.93 (2.63-6.02)**	**8.39E-11**	**4.20 (2.75-6.57)**	**9.10E-11**	**2.54 (1.49-4.41)**	**7.04E-04**
FGF-23		0.97 (0.70-1.30)	8.30E-01	0.95 (0.68-1.31)	7.76E-01	1.61 (1.08-2.38)	1.76E-02
LIF-R		1.11 (0.63-1.96)	7.13E-01	1.04 (0.58-1.86)	8.99E-01	0.99 (0.45-2.16)	9.78E-01
FGF-21		1.10 (0.93-1.29)	2.59E-01	1.10 (0.93-1.30)	2.78E-01	1.23 (0.97-1.58)	8.66E-02
CCL19		1.01 (0.80-1.25)	9.46E-01	1.01 (0.80-1.28)	9.05E-01	1.06 (0.77-1.44)	7.31E-01
IL-10RB		1.06 (0.67-1.68)	8.02E-01	1.04 (0.65-1.65)	8.83E-01	1.05 (0.56-1.97)	8.78E-01
IL-18R1		1.75 (1.14-2.70)	1.13E-02	1.72 (1.10-2.71)	1.86E-02	1.00 (0.53-1.88)	9.88E-01
CXCL5		1.03 (0.91-1.16)	6.52E-01	1.04 (0.92-1.18)	5.37E-01	1.06 (0.89-1.26)	5.24E-01
TRANCE	x	**0.42 (0.31-0.56)**	**1.35E-08**	0.39 (0.29-0.53)	2.71E-09	0.65 (0.43-0.97)	3.72E-02
HGF	x	**1.82 (1.44-2.32)**	**7.33E-07**	1.78 (1.40-2.28)	3.17E-06	1.40 (1.00-1.94)	4.87E-02
IL-12B		0.79 (0.59-1.06)	1.14E-01	0.81 (0.60-1.10)	1.73E-01	0.92 (0.62-1.36)	6.77E-01
MMP-10		0.87 (0.66-1.15)	3.42E-01	0.89 (0.66-1.17)	4.15E-01	0.97 (0.67-1.39)	8.74E-01
IL-10	x	**1.56 (1.20-2.06)**	**1.06E-03**	1.56 (1.20-2.07)	1.14E-03	1.36 (0.97-1.89)	6.68E-02
CCL23	x	**3.11 (2.02-4.87)**	**4.38E-07**	**3.35 (2.14-5.37)**	**2.58E-07**	1.40 (0.77-2.53)	2.73E-01
CD5		0.46 (0.27-0.77)	3.79E-03	0.44 (0.25-0.75)	3.30E-03	0.69 (0.33-1.41)	3.15E-01
CCL3		1.51 (1.04-2.20)	2.90E-02	1.51 (1.03-2.21)	3.56E-02	1.47 (0.86-2.46)	1.52E-01
Flt3L	x	**0.38 (0.25-0.56)**	**2.60E-06**	**0.35 (0.22-0.53)**	**1.66E-06**	0.67 (0.39-1.14)	1.40E-01
CXCL6		1.15 (0.89-1.49)	2.89E-01	1.15 (0.88-1.51)	3.09E-01	1.32 (0.91-1.90)	1.41E-01
CXCL10		1.28 (1.03-1.58)	2.21E-02	1.30 (1.04-1.61)	1.82E-02	1.43 (1.09-1.90)	1.16E-02
4E-BP1	x	**1.36 (1.16-1.58)**	**1.03E-04**	**1.33 (1.14-1.56)**	**3.91E-04**	**1.54 (1.24-1.94)**	**1.46E-04**
SIRT2	x	**1.33 (1.16-1.53)**	**4.82E-05**	**1.34 (1.16-1.55)**	**8.62E-05**	**1.44 (1.18-1.77)**	**4.12E-04**
CCL28		1.39 (0.92-2.09)	1.14E-01	1.33 (0.86-2.02)	1.89E-01	1.15 (0.66-1.99)	6.11E-01
DNER	x	**0.22 (0.11-0.42)**	**5.58E-06**	**0.21 (0.11-0.41)**	**7.71E-06**	0.37 (0.15-0.89)	2.87E-02
EN-RAGE	x	**2.03 (1.63-2.55)**	**4.53E-10**	**2.04 (1.62-2.58)**	**1.69E-09**	1.42 (1.04-1.93)	2.45E-02
CD40		1.72 (1.12-2.64)	1.26E-02	1.84 (1.17-2.92)	8.60E-03	1.74 (0.93-3.20)	7.82E-02
IFN-gamma		0.73 (0.46-1.11)	1.69E-01	0.75 (0.46-1.14)	2.08E-01	0.83 (0.45-1.41)	5.21E-01
FGF-19		1.28 (1.03-1.60)	2.95E-02	1.29 (1.04-1.62)	2.38E-02	1.18 (0.87-1.61)	2.89E-01
MCP-2		0.97 (0.71-1.32)	8.42E-01	0.97 (0.70-1.34)	8.57E-01	1.12 (0.72-1.74)	6.15E-01
CASP-8	x	**1.79 (1.31-2.46)**	**2.67E-04**	1.73 (1.24-2.43)	1.25E-03	1.91 (1.21-2.98)	4.37E-03
CCL25	x	**0.46 (0.33-0.64)**	**3.81E-06**	**0.42 (0.29-0.58)**	**7.69E-07**	0.54 (0.34-0.84)	6.85E-03
CX3CL1		1.20 (0.77-1.84)	4.16E-01	1.13 (0.72-1.76)	5.93E-01	1.40 (0.77-2.52)	2.65E-01
TNFRSF9		0.61 (0.39-0.92)	1.99E-02	0.57 (0.36-0.87)	1.14E-02	0.66 (0.37-1.16)	1.54E-01
NT-3		1.16 (0.80-1.67)	4.13E-01	1.11 (0.76-1.60)	5.75E-01	1.09 (0.68-1.70)	6.89E-01
TWEAK		0.59 (0.37-0.91)	1.79E-02	0.56 (0.35-0.88)	1.29E-02	0.91 (0.51-1.59)	7.33E-01
CCL20		1.29 (1.06-1.56)	8.99E-03	1.31 (1.08-1.59)	6.32E-03	1.23 (0.94-1.60)	1.20E-01
STAMPB	x	**1.41 (1.18-1.70)**	**2.18E-04**	1.42 (1.17-1.73)	3.29E-04	**1.59 (1.22-2.09)**	**6.02E-04**
ADA		1.27 (0.85-1.86)	2.31E-01	1.33 (0.87-2.03)	1.83E-01	1.38 (0.77-2.44)	2.72E-01
TNFB		0.54 (0.36-0.79)	1.56E-03	0.53 (0.35-0.79)	2.27E-03	0.64 (0.38-1.08)	1.30E-01
CSF-1	x	**5.06 (2.78-9.50)**	**2.24E-07**	5.06 (2.72-9.69)	5.39E-07	1.99 (0.89-4.62)	9.97E-02

OR<1 and *P* <0.05	OR >1 and *P* <0.05	*P* >0.05

Model 1: age, sex, day of blood draw and diabetes mellitus; Model 2: age, sex, day of blood draw, diabetes mellitus and stroke severity (maximum NIHSS score). OR, odds ratio

Table S3: Area under the receiver operating characteristics curve (AUC) for the LASSO model and for the individual proteins included in the model for all ischemic stroke and for sex-stratified analyses.

	All Ischemic stroke				Sex-stratified							
					Males				Females			
Order of importance		univ	AUC [95% CI] protein only	AUC [95% CI] protein + NIHSS		univ	AUC [95% CI] protein only	AUC [95% CI] protein + NIHSS		univ	AUC [95% CI] protein only	AUC [95% CI] protein + NIHSS
	max NIHSS		-	0.91 [0.88-0.94]	max NIHSS		-	0.89 [0.85-0.93]	max NIHSS		-	0.94 [0.90-0.98]
1	TRAIL		0.68 [0.62-0.73]	0.91 [0.88-0.94]	TRAIL		0.72 [0.65-0.78]	0.90 [0.86-0.94]	Flt3L		0.68 [0.59-0.77]	0.94 [0.91-0.98]
2	EN-RAGE		0.71 [0.66-0.77]	0.91 [0.88-0.94]	IL-6		0.75 [0.69-0.81]	0.90 [0.86-0.94]	IL-10RB		0.65 [0.55-0.76]	0.94 [0.90-0.98]
3	IL-6		0.75 [0.70-0.80]	0.91 [0.88-0.94]	OSM		0.70 [0.64-0.77]	0.91 [0.88-0.94]	EN-RAGE		0.72 [0.62-0.82]	0.94 [0.89-0.98]
4	OSM		0.69 [0.63-0.74]	0.92 [0.90-0.94]	EN-RAGE		0.71 [0.65-0.78]	0.90 [0.86-0.93]	SCF		0.62 [0.52-0.72]	0.94 [0.90-0.98]
5	Flt3L		0.64 [0.59-0.70]	0.91 [0.88-0.94]	TNFSF14		0.72 [0.66-0.78]	0.90 [0.87-0.94]	CSF-1		0.73 [0.64-0.82]	0.94 [0.90-0.98]
6	TNFSF14		0.71 [0.66-0.76]	0.91 [0.89-0.94]	CCL25		0.64 [0.57-0.71]	0.90 [0.86-0.94]	GDNF		0.64 [0.54-0.74]	0.95 [0.91-0.98]
7	CSF-1		0.66 [0.61-0.72]	0.91 [0.87-0.94]	CSF-1		0.64 [0.57-0.71]	0.89 [0.85-0.93]	uPA		0.61 [0.51-0.71]	0.94 [0.90-0.98]
8	CCL25		0.64 [0.58-0.69]	0.91 [0.88-0.94]	-	-	-	-	SLAMF1		0.63 [0.53-0.73]	0.94 [0.90-0.98]
9	HGF		0.68 [0.63-0.74]	0.91 [0.88-0.94]	-	-	-	-	TNFSF14		0.70 [0.60-0.80]	0.94 [0.91-0.98]
10	-	-	-	-	-	-	-	-	-	-	-	-
LASSO	multi-protein		0.81 [0.76-0.86]	0.92 [0.90-0.95]	multi-protein		0.82 [0.76-0.87]	0.96 [0.91-1]	multi-protein		0.86 [0.79-0.93]	0.95 [0.91-0.99]

OR<1 and *P*<0.05 in univariable regression

OR >1 and *P*<0.05 univariable regression

Table S3, Continued: Area under the receiver operating characteristics curve (AUC) for the LASSO model and for the individual proteins included in the model for all ischemic stroke and for TOAST-stratified analyses.

	Large artery atherosclerosis				Small Artery Occlusion			
Order of importance		univ	AUC [95% CI] protein only	AUC [95% CI] protein + NIHSS	Protein Name	univ	AUC [95% CI] protein only	AUC [95% CI] protein + NIHSS
	max NIHSS		-	0.88 [0.79-0.97]	max NIHSS		-	0.90 [0.78-1.00]
1	IL-6		0.78 [0.66-0.89]	0.91 [0.84-0.98]	GDNF		0.74 [0.56-0.92]	0.89 [0.74-1.00]
2	EN-RAGE		0.78 [0.66-0.90]	0.89 [0.82-0.97]	TNFB		0.69 [0.53-0.84]	0.91 [0.78-1.00]
LASSO	multi-protein		0.79 [0.68-0.90]	0.91 [0.83-0.98]	multi-protein		0.82 [0.67-0.97]	0.91 [0.76-1]

	Cardioembolic Stroke				Cryptogenic Stroke			
Order of importance	Protein Name	univ	AUC [95% CI] protein only	AUC [95% CI] protein + NIHSS	Protein Name	univ	AUC [95% CI] protein only	AUC [95% CI] protein + NIHSS
			-	0.90 [0.84-0.96]			-	0.93 [0.88-0.99]
1	TRAIL		0.74 [0.62-0.85]	0.91 [0.85-0.97]	EN-RAGE		0.76 [0.65-0.88]	0.94 [0.90-0.99]
2	EN-RAGE		0.80 [0.70-0.89]	0.91 [0.86-0.97]	CD6		0.65 [0.53-0.77]	0.93 [0.88-0.99]
3	MCP-3		0.77 [0.67-0.86]	0.90 [0.84-0.96]	CCL25		0.67 [0.56-0.79]	0.93 [0.88-0.99]
4	IL-10		0.73 [0.61-0.84]	0.90 [0.84-0.96]	TNFSF14		0.72 [0.61-0.82]	0.94 [0.89-0.99]
5	SIRT2		0.74 [0.62-0.86]	0.91 [0.85-0.97]	MCP-3		0.73 [0.62-0.83]	0.95 [0.90-0.99]
6	Flt3L		0.70 [0.58-0.82]	0.91 [0.85-0.97]	CASP-8		0.67 [0.55-0.78]	0.95 [0.91-0.99]
7	HGF		0.76 [0.66-0.86]	0.91 [0.85-0.97]	NT-3		0.60 [0.49-0.72]	0.93 [0.88-0.98]
8	GDNF		0.62 [0.50-0.75]	0.90 [0.84-0.96]	TRANCE		0.65 [0.54-0.77]	0.93 [0.87-0.99]
9	IL-8		0.77 [0.67-0.87]	0.91 [0.85-0.97]	CDCP1		0.66 [0.54-0.78]	0.94 [0.89-0.99]
10	CCL4		0.67 [0.54-0.79]	0.90 [0.84-0.97]	-	-	-	-
LASSO	multi-protein		0.93 [0.87-1]	0.97 [0.93-1]	multi-protein		0.89 [0.81-0.97]	0.98 [0.96-1]

OR<1 and *P*<0.05 in univariable regression

OR >1 and *P*<0.05 univariable regression

Table S4: Odds ratios and 95% confidence intervals for poor outcome per doubling of protein levels as compared to good outcome stratified by sex (univariable).

Protein Name	Sex-stratified (univariate)			
	Women_OR	Women_p.value	Men_OR	Men_p.value
IL-8	1.31 (0.83-2.02)	2.28E-01	1.64 (1.21-2.27)	1.99E-03
VEGF-A	2.33 (1.06-5.28)	3.63E-02	1.93 (1.14-3.30)	1.47E-02
MCP-3	2.13 (1.25-3.73)	6.00E-03	2.06 (1.45-2.97)	6.84E-05
GDNF	3.10 (1.37-7.42)	7.98E-03	1.65 (0.95-2.90)	7.94E-02
CDCP1	1.67 (0.94-3.00)	8.24E-02	1.19 (0.80-1.75)	3.90E-01
CD244	0.84 (0.31-2.21)	7.31E-01	0.93 (0.48-1.76)	8.29E-01
IL-7	1.53 (0.95-2.45)	7.69E-02	1.28 (0.93-1.75)	1.28E-01
OPG	1.58 (0.64-3.95)	3.22E-01	1.86 (1.03-3.41)	4.16E-02
LAP TGF-beta-1	1.68 (0.89-3.13)	1.04E-01	1.41 (0.88-2.23)	1.46E-01
uPA	0.28 (0.09-0.79)	1.86E-02	0.48 (0.24-0.93)	3.43E-02
IL-6	1.88 (1.34-2.71)	4.43E-04	1.98 (1.59-2.52)	4.32E-09
MCP-1	1.37 (0.64-2.96)	4.15E-01	1.08 (0.66-1.73)	7.65E-01
CXCL11	1.10 (0.78-1.54)	5.65E-01	1.12 (0.88-1.42)	3.46E-01
AXIN1	1.26 (0.94-1.68)	1.24E-01	1.25 (1.04-1.51)	1.71E-02
TRAIL	0.38 (0.16-0.85)	2.16E-02	0.12 (0.06-0.23)	5.40E-09
CXCL9	0.97 (0.66-1.40)	8.89E-01	0.97 (0.73-1.27)	8.31E-01
CST5	1.03 (0.49-2.15)	9.28E-01	0.55 (0.32-0.93)	3.01E-02
OSM	1.71 (1.20-2.49)	3.65E-03	2.08 (1.62-2.72)	2.96E-08
CXCL1	1.17 (0.87-1.60)	3.01E-01	1.15 (0.93-1.43)	2.01E-01
CCL4	1.77 (0.98-3.20)	5.75E-02	1.28 (0.87-1.87)	2.07E-01
CD6	0.71 (0.37-1.35)	3.11E-01	0.43 (0.26-0.71)	1.01E-03
SCF	0.51 (0.27-0.96)	3.54E-02	0.50 (0.31-0.77)	2.29E-03
IL-18	1.33 (0.76-2.32)	3.17E-01	0.89 (0.57-1.38)	5.98E-01
SLAMF1	2.46 (1.21-5.14)	1.40E-02	1.07 (0.69-1.65)	7.44E-01
MCP-4	1.20 (0.70-2.06)	5.04E-01	0.72 (0.47-1.06)	1.08E-01
CCL11	0.52 (0.25-1.05)	7.56E-02	0.73 (0.45-1.17)	1.98E-01
TNFSF14	4.63 (2.12-10.72)	1.86E-04	3.75 (2.36-6.20)	7.96E-08
FGF-23	1.40 (0.88-2.22)	1.47E-01	0.76 (0.49-1.15)	2.20E-01
LIF-R	0.98 (0.34-2.80)	9.69E-01	1.18 (0.60-2.32)	6.27E-01
FGF-21	1.11 (0.83-1.47)	4.88E-01	1.12 (0.92-1.37)	2.54E-01
CCL19	0.96 (0.65-1.37)	8.14E-01	1.07 (0.80-1.41)	6.51E-01
IL-10RB	2.91 (1.21-7.36)	1.97E-02	0.72 (0.41-1.24)	2.38E-01
IL-18R1	1.85 (0.85-4.06)	1.19E-01	1.74 (1.03-2.95)	3.73E-02
CXCL5	1.04 (0.85-1.28)	6.96E-01	1.04 (0.90-1.21)	6.03E-01
TRANCE	0.50 (0.28-0.84)	1.12E-02	0.38 (0.26-0.55)	3.02E-07
HGF	2.04 (1.33-3.24)	1.51E-03	1.72 (1.30-2.29)	1.79E-04
IL-12B	0.97 (0.58-1.64)	9.22E-01	0.75 (0.52-1.07)	1.16E-01
MMP-10	0.83 (0.50-1.34)	4.62E-01	0.92 (0.65-1.27)	6.06E-01
IL-10	1.45 (0.98-2.18)	5.93E-02	1.68 (1.18-2.46)	5.51E-03
CCL23	3.80 (1.74-8.78)	1.13E-03	2.84 (1.69-4.87)	1.01E-04
CD5	0.95 (0.40-2.20)	9.14E-01	0.32 (0.16-0.62)	1.08E-03
CCL3	2.03 (1.06-3.92)	3.12E-02	1.30 (0.82-2.05)	2.56E-01
Flt3L	0.23 (0.10-0.48)	1.96E-04	0.48 (0.29-0.77)	2.60E-03
CXCL6	1.19 (0.76-1.86)	4.35E-01	1.16 (0.84-1.60)	3.70E-01
CXCL10	1.19 (0.83-1.69)	3.42E-01	1.36 (1.04-1.78)	2.37E-02
4E-BP1	1.35 (1.03-1.77)	2.93E-02	1.36 (1.13-1.64)	1.36E-03
SIRT2	1.32 (1.03-1.69)	2.53E-02	1.34 (1.14-1.59)	6.08E-04
CCL28	1.50 (0.70-3.13)	2.84E-01	1.40 (0.85-2.28)	1.80E-01
DNER	0.24 (0.07-0.72)	1.36E-02	0.20 (0.09-0.44)	9.21E-05
EN-RAGE	2.35 (1.56-3.67)	8.82E-05	1.90 (1.47-2.48)	1.47E-06
CD40	2.27 (1.06-4.91)	3.44E-02	1.54 (0.91-2.60)	1.02E-01
IFN-gamma	0.50 (0.19-1.05)	1.13E-01	0.93 (0.53-1.53)	7.90E-01
FGF-19	1.42 (0.96-2.14)	8.36E-02	1.23 (0.94-1.61)	1.36E-01
MCP-2	1.23 (0.69-2.24)	4.88E-01	0.89 (0.62-1.29)	5.42E-01
CASP-8	1.71 (0.90-3.23)	9.82E-02	1.80 (1.26-2.62)	1.49E-03
CCL25	0.45 (0.25-0.78)	5.24E-03	0.46 (0.30-0.69)	2.21E-04
CX3CL1	1.37 (0.65-2.83)	4.02E-01	1.15 (0.66-1.95)	6.21E-01
TNFRSF9	1.08 (0.55-2.07)	8.18E-01	0.42 (0.24-0.73)	2.62E-03
NT-3	0.48 (0.19-1.10)	1.03E-01	1.55 (1.00-2.47)	5.48E-02
TWEAK	0.76 (0.34-1.63)	4.95E-01	0.52 (0.30-0.88)	1.76E-02
CCL20	1.22 (0.85-1.71)	2.59E-01	1.34 (1.06-1.69)	1.31E-02
STAMPB	1.40 (1.00-1.96)	5.08E-02	1.42 (1.14-1.77)	1.84E-03
ADA	1.44 (0.71-2.87)	3.05E-01	1.17 (0.71-1.86)	5.22E-01
TNFB	0.55 (0.28-1.06)	7.77E-02	0.55 (0.34-0.89)	1.58E-02
CSF-1	9.70 (2.85-36.29)	4.36E-04	4.10 (2.08-8.42)	7.24E-05

OR<1 and *P*<0.05	OR >1 and *P*<0.05	*P*>0.05

Table S4, Continued: Odds ratios and 95% confidence intervals for poor outcome per doubling of protein levels as compared to good outcome stratified by TOAST subtype (univariable).

Protein Name	TOAST subtype-stratified (univariate)							
	LAA_OR	LAA_p.value	SAO_OR	SAO_p.value	CE_OR	CE_p.value	Crypt_OR	Crypt_p.value
IL-8	1.53 (0.77-3.13)	2.24E-01	1.14 (0.46-2.49)	7.58E-01	5.89 (2.36-17.00)	3.76E-04	1.26 (0.76-2.05)	3.34E-01
VEGF-A	1.91 (0.62-6.38)	2.61E-01	1.14 (0.23-4.46)	8.60E-01	6.51 (1.86-26.95)	5.41E-03	2.35 (0.87-6.56)	9.25E-02
MCP-3	2.18 (0.91-5.79)	9.16E-02	0.67 (0.17-1.80)	4.95E-01	4.01 (1.91-9.43)	5.48E-04	2.42 (1.28-4.90)	9.13E-03
GDNF	1.04 (0.33-3.23)	9.44E-01	11.44 (2.05-81.96)	8.68E-03	3.05 (1.10-9.53)	4.03E-02	1.53 (0.60-4.00)	3.69E-01
CDCP1	1.53 (0.65-3.65)	3.26E-01	0.65 (0.14-2.49)	5.49E-01	0.93 (0.46-1.82)	8.31E-01	2.26 (1.16-4.59)	1.87E-02
CD244	0.79 (0.15-4.11)	7.76E-01	0.37 (0.05-2.42)	3.19E-01	1.32 (0.40-4.34)	6.49E-01	1.01 (0.30-3.22)	9.87E-01
IL-7	1.02 (0.49-2.04)	9.58E-01	1.13 (0.41-2.74)	8.05E-01	1.90 (1.11-3.42)	2.41E-02	1.56 (0.91-2.67)	9.73E-02
OPG	2.28 (0.57-9.89)	2.52E-01	1.30 (0.22-8.16)	7.74E-01	1.76 (0.64-4.82)	2.67E-01	2.39 (0.83-7.18)	1.11E-01
LAP TGF-beta-1	1.50 (0.47-4.88)	4.87E-01	0.94 (0.19-3.40)	9.32E-01	1.69 (0.73-4.02)	2.17E-01	1.38 (0.64-2.91)	4.03E-01
uPA	0.43 (0.08-2.04)	2.92E-01	0.22 (0.02-1.72)	1.57E-01	0.60 (0.19-1.60)	3.43E-01	0.36 (0.09-1.25)	1.15E-01
IL-6	2.99 (1.58-6.52)	2.20E-03	1.01 (0.44-1.87)	9.86E-01	2.29 (1.51-3.74)	2.86E-04	1.63 (1.15-2.43)	1.00E-02
MCP-1	0.79 (0.27-2.22)	6.59E-01	1.75 (0.44-6.63)	4.10E-01	1.46 (0.49-4.45)	4.92E-01	1.02 (0.42-2.37)	9.67E-01
CXCL11	1.13 (0.60-2.16)	6.96E-01	0.82 (0.34-1.89)	6.54E-01	1.39 (0.94-2.08)	1.02E-01	1.07 (0.67-1.70)	7.60E-01
AXIN1	1.04 (0.67-1.62)	8.48E-01	0.90 (0.44-1.65)	7.44E-01	1.85 (1.24-2.94)	4.85E-03	1.15 (0.82-1.59)	4.12E-01
TRAIL	0.28 (0.06-1.11)	8.44E-02	0.37 (0.04-3.28)	3.90E-01	0.14 (0.04-0.43)	1.16E-03	0.30 (0.10-0.87)	2.93E-02
CXCL9	0.94 (0.50-1.68)	8.41E-01	1.16 (0.54-2.24)	6.79E-01	1.05 (0.66-1.63)	8.22E-01	0.84 (0.51-1.32)	4.69E-01
CST5	0.55 (0.17-1.56)	2.80E-01	1.27 (0.35-4.19)	7.01E-01	1.34 (0.49-3.70)	5.67E-01	0.44 (0.17-1.08)	8.02E-02
OSM	1.57 (0.95-2.74)	9.43E-02	1.57 (0.82-3.13)	1.80E-01	2.35 (1.44-4.10)	1.22E-03	2.01 (1.32-3.18)	1.63E-03
CXCL1	1.12 (0.67-1.94)	6.70E-01	0.95 (0.55-1.61)	8.40E-01	1.25 (0.84-1.90)	2.72E-01	1.09 (0.77-1.56)	6.13E-01
CCL4	1.00 (0.39-2.46)	9.98E-01	1.36 (0.46-3.49)	5.47E-01	2.92 (1.27-7.35)	1.53E-02	1.27 (0.69-2.29)	4.35E-01
CD6	0.35 (0.11-0.96)	5.27E-02	0.38 (0.08-1.54)	1.92E-01	0.42 (0.17-0.92)	3.91E-02	0.39 (0.16-0.89)	2.99E-02
SCF	0.38 (0.13-0.98)	5.29E-02	1.11 (0.27-5.65)	8.96E-01	0.45 (0.19-1.03)	6.20E-02	0.50 (0.21-1.15)	9.72E-02
IL-18	0.69 (0.26-1.83)	4.52E-01	0.61 (0.15-2.15)	4.68E-01	1.10 (0.49-2.43)	8.18E-01	1.43 (0.70-2.95)	3.27E-01
SLAMF1	1.42 (0.47-4.51)	5.41E-01	0.94 (0.24-3.16)	9.28E-01	1.32 (0.64-2.67)	4.39E-01	1.44 (0.59-3.57)	4.20E-01
MCP-4	0.50 (0.18-1.20)	1.43E-01	1.87 (0.73-4.75)	1.82E-01	0.94 (0.45-1.93)	8.58E-01	0.95 (0.49-1.72)	8.60E-01
CCL11	0.71 (0.25-1.86)	4.96E-01	0.94 (0.23-3.46)	9.28E-01	1.09 (0.45-2.60)	8.54E-01	0.78 (0.34-1.72)	5.51E-01
TNFSF14	4.49 (1.60-15.01)	7.66E-03	1.03 (0.20-4.66)	9.73E-01	4.71 (1.82-14.02)	2.61E-03	5.19 (2.08-14.28)	7.08E-04
FGF-23	0.64 (0.18-1.66)	4.28E-01	2.92 (0.95-9.05)	5.65E-02	1.23 (0.69-2.16)	4.61E-01	0.81 (0.39-1.57)	5.51E-01
LIF-R	3.39 (0.48-26.39)	2.25E-01	0.78 (0.11-4.93)	7.99E-01	1.60 (0.45-5.90)	4.69E-01	0.84 (0.27-2.58)	7.55E-01
FGF-21	1.15 (0.75-1.76)	5.02E-01	0.96 (0.54-1.74)	8.89E-01	1.32 (0.94-1.91)	1.18E-01	0.98 (0.70-1.37)	9.10E-01
CCL19	1.06 (0.56-1.94)	8.51E-01	0.56 (0.20-1.30)	2.31E-01	0.83 (0.48-1.38)	4.88E-01	1.16 (0.69-1.92)	5.56E-01
IL-10RB	1.49 (0.38-6.12)	5.67E-01	2.18 (0.57-7.76)	2.25E-01	0.86 (0.24-2.98)	8.04E-01	1.30 (0.47-3.55)	6.05E-01
IL-18R1	3.70 (0.95-16.51)	6.81E-02	0.29 (0.04-1.61)	1.74E-01	1.18 (0.41-3.35)	7.51E-01	1.69 (0.69-4.19)	2.55E-01
CXCL5	0.96 (0.68-1.37)	8.30E-01	0.86 (0.61-1.21)	3.92E-01	1.23 (0.93-1.66)	1.49E-01	1.04 (0.82-1.33)	7.56E-01
TRANCE	0.45 (0.20-0.93)	3.52E-02	0.99 (0.31-3.15)	9.89E-01	0.55 (0.27-1.06)	8.13E-02	0.42 (0.22-0.76)	5.40E-03
HGF	1.27 (0.68-2.33)	4.30E-01	1.82 (0.79-3.85)	1.17E-01	2.37 (1.41-4.23)	1.89E-03	1.64 (0.99-2.74)	4.97E-02
IL-12B	0.90 (0.38-2.05)	7.98E-01	0.77 (0.30-1.93)	5.73E-01	0.72 (0.37-1.38)	3.25E-01	0.92 (0.48-1.71)	7.84E-01
MMP-10	0.94 (0.49-1.74)	8.44E-01	1.92 (0.62-6.20)	2.60E-01	1.02 (0.51-1.89)	9.50E-01	0.64 (0.35-1.11)	1.27E-01
IL-10	2.97 (1.14-11.36)	7.19E-02	0.95 (0.29-1.77)	8.97E-01	2.97 (1.46-6.68)	4.31E-03	1.28 (0.69-2.30)	4.02E-01
CCL23	2.64 (0.75-10.44)	1.42E-01	1.58 (0.28-8.70)	5.97E-01	4.13 (1.57-12.44)	6.50E-03	3.23 (1.30-8.51)	1.38E-02
CD5	0.42 (0.08-1.66)	2.47E-01	0.80 (0.14-3.78)	7.94E-01	0.31 (0.08-1.00)	6.22E-02	0.43 (0.13-1.36)	1.57E-01
CCL3	1.38 (0.51-3.78)	5.18E-01	1.68 (0.46-5.38)	4.00E-01	2.53 (1.00-7.11)	6.02E-02	0.91 (0.40-1.94)	8.11E-01
Flt3L	0.71 (0.23-2.18)	5.47E-01	0.48 (0.11-2.00)	3.18E-01	0.27 (0.10-0.63)	4.27E-03	0.42 (0.17-0.99)	5.36E-02
CXCL6	0.86 (0.41-1.72)	6.74E-01	0.95 (0.37-2.32)	9.12E-01	1.40 (0.85-2.37)	1.89E-01	1.20 (0.68-2.12)	5.36E-01
CXCL10	1.43 (0.78-2.69)	2.51E-01	1.17 (0.55-2.30)	6.57E-01	1.16 (0.70-1.93)	5.57E-01	1.31 (0.82-2.06)	2.52E-01
4E-BP1	1.02 (0.70-1.47)	9.19E-01	1.39 (0.80-2.36)	2.19E-01	1.53 (1.08-2.22)	1.95E-02	1.44 (1.07-1.97)	1.72E-02
SIRT2	1.11 (0.78-1.59)	5.66E-01	1.14 (0.65-1.88)	6.34E-01	1.82 (1.30-2.67)	9.60E-04	1.33 (1.01-1.76)	4.29E-02
CCL28	1.79 (0.56-5.90)	3.20E-01	2.12 (0.55-7.02)	2.28E-01	2.18 (0.89-5.91)	9.69E-02	1.45 (0.61-3.27)	3.73E-01
DNER	0.39 (0.07-1.92)	2.55E-01	0.21 (0.02-2.05)	1.84E-01	0.14 (0.03-0.58)	1.02E-02	0.32 (0.07-1.31)	1.20E-01
EN-RAGE	2.70 (1.45-5.71)	3.81E-03	1.27 (0.52-2.70)	5.63E-01	2.41 (1.54-4.06)	3.18E-04	3.30 (1.89-6.24)	7.45E-05
CD40	1.48 (0.46-4.73)	4.97E-01	2.27 (0.47-10.71)	2.93E-01	2.62 (1.08-6.94)	3.92E-02	1.73 (0.64-4.61)	2.74E-01
IFN-gamma	1.43 (0.59-3.69)	4.05E-01	0.37 (0.04-1.67)	3.07E-01	0.62 (0.22-1.37)	2.94E-01	0.73 (0.24-1.69)	5.19E-01
FGF-19	2.13 (1.19-4.08)	1.52E-02	1.08 (0.53-2.20)	8.21E-01	1.29 (0.80-2.11)	2.98E-01	1.48 (0.90-2.52)	1.32E-01
MCP-2	1.21 (0.51-2.93)	6.62E-01	1.26 (0.42-4.30)	6.97E-01	1.08 (0.56-2.12)	8.18E-01	0.97 (0.47-2.04)	9.34E-01
CASP-8	1.04 (0.53-1.91)	9.07E-01	1.36 (0.36-4.42)	6.27E-01	3.38 (1.49-8.59)	6.00E-03	3.13 (1.42-7.28)	5.59E-03
CCL25	0.49 (0.18-1.19)	1.33E-01	0.77 (0.26-2.09)	6.15E-01	0.57 (0.26-1.18)	1.34E-01	0.34 (0.15-0.70)	4.74E-03
CX3CL1	1.33 (0.46-3.76)	5.88E-01	2.09 (0.67-6.08)	1.72E-01	1.31 (0.44-3.93)	6.26E-01	1.48 (0.53-4.11)	4.51E-01
TNFRSF9	0.37 (0.11-1.08)	8.85E-02	1.30 (0.36-4.02)	6.62E-01	0.90 (0.31-2.51)	8.39E-01	0.51 (0.19-1.27)	1.68E-01
NT-3	3.00 (1.14-11.49)	7.64E-02	1.66 (0.35-7.34)	5.08E-01	1.33 (0.46-3.85)	5.97E-01	0.35 (0.12-0.90)	4.35E-02
TWEAK	0.58 (0.16-1.83)	3.61E-01	1.54 (0.29-7.25)	5.97E-01	0.78 (0.31-1.83)	5.88E-01	0.48 (0.18-1.17)	1.20E-01
CCL20	1.43 (0.79-2.67)	2.29E-01	1.10 (0.49-2.15)	7.99E-01	1.69 (0.98-3.14)	6.86E-02	1.39 (0.97-1.99)	7.08E-02
STAMPB	1.02 (0.63-1.63)	9.40E-01	1.18 (0.54-2.39)	6.56E-01	2.07 (1.33-3.45)	2.47E-03	1.40 (0.96-2.05)	7.84E-02
ADA	0.97 (0.32-2.62)	9.48E-01	1.21 (0.24-5.58)	8.06E-01	1.48 (0.60-3.64)	3.82E-01	2.46 (0.97-6.40)	5.76E-02
TNFB	1.15 (0.52-2.61)	7.20E-01	0.32 (0.08-1.19)	8.94E-02	0.66 (0.26-1.54)	3.48E-01	0.52 (0.23-1.15)	1.16E-01
CSF-1	10.29 (2.04-70.15)	8.50E-03	3.41 (0.38-31.31)	2.68E-01	4.68 (1.18-21.24)	3.48E-02	3.49 (0.98-13.17)	5.70E-02

OR<1 and *P* <0.05	OR >1 and *P* <0.05	*P* >0.05

LAA, large artery atherosclerosis; SAO, small artery occlusion; CE, cardioembolic stroke; Crypt, cryptogenic stroke

Table S5: Odds ratios and 95% confidence intervals for poor outcome per doubling of protein levels as compared to good outcome in sensitivity analyses (univariable).

Protein Name	FDR significant All_Univariate	Infection excluded (univariate)		Recurrent event excluded (univariate)	
		Odds ratio	*P*-value	Odds ratio	*P*-value
IL-8		1.44 (1.10-1.89)	7.1E-03	1.48 (1.14-1.91)	2.9E-03
VEGF-A		1.96 (1.22-3.18)	5.8E-03	1.97 (1.25-3.12)	3.6E-03
MCP-3	x	2.07 (1.52-2.84)	4.8E-06	2.02 (1.49-2.75)	6.6E-06
GDNF		2.12 (1.33-3.44)	1.8E-03	2.14 (1.33-3.48)	1.8E-03
CDCP1		1.36 (0.98-1.91)	6.8E-02	1.41 (1.01-1.98)	4.3E-02
CD244		1.16 (0.65-2.05)	6.2E-01	0.91 (0.52-1.58)	7.4E-01
IL-7		1.39 (1.06-1.83)	1.8E-02	1.35 (1.03-1.77)	2.8E-02
OPG		1.49 (0.88-2.51)	1.4E-01	1.76 (1.04-2.97)	3.4E-02
LAP TGF-beta-1		1.58 (1.06-2.35)	2.2E-02	1.46 (0.99-2.14)	5.2E-02
uPA		0.43 (0.23-0.78)	6.1E-03	0.47 (0.25-0.84)	1.3E-02
IL-6	x	2.13 (1.72-2.66)	1.2E-11	1.86 (1.54-2.27)	4.7E-10
MCP-1		1.09 (0.71-1.66)	7.0E-01	1.18 (0.77-1.80)	4.4E-01
CXCL11		1.09 (0.87-1.34)	4.5E-01	1.11 (0.91-1.35)	3.0E-01
AXIN1		1.31 (1.11-1.56)	1.4E-03	1.22 (1.04-1.43)	1.5E-02
TRAIL	x	0.21 (0.12-0.37)	5.4E-08	0.21 (0.12-0.36)	1.4E-08
CXCL9		0.98 (0.78-1.23)	8.8E-01	0.96 (0.76-1.21)	7.5E-01
CST5		0.59 (0.37-0.91)	2.0E-02	0.68 (0.43-1.06)	8.9E-02
OSM	x	1.97 (1.59-2.47)	1.9E-09	1.96 (1.58-2.45)	2.0E-09
CXCL1		1.13 (0.95-1.36)	1.8E-01	1.15 (0.96-1.38)	1.3E-01
CCL4		1.44 (1.04-2.01)	2.9E-02	1.50 (1.08-2.09)	1.6E-02
CD6	x	0.58 (0.38-0.87)	1.0E-02	0.53 (0.35-0.80)	2.6E-03
SCF	x	0.53 (0.35-0.78)	1.6E-03	0.53 (0.36-0.77)	9.1E-04
IL-18		1.08 (0.76-1.54)	6.6E-01	1.10 (0.77-1.57)	6.0E-01
SLAMF1		1.37 (0.94-2.00)	9.6E-02	1.34 (0.91-1.96)	1.4E-01
MCP-4		0.94 (0.67-1.29)	6.9E-01	0.90 (0.64-1.24)	5.2E-01
CCL11		0.74 (0.49-1.11)	1.5E-01	0.68 (0.45-1.04)	7.8E-02
TNFSF14	x	4.38 (2.8-7.00)	2.3E-10	3.80 (2.50-5.94)	1.5E-09
FGF-23		0.97 (0.69-1.32)	8.4E-01	0.98 (0.70-1.34)	9.1E-01
LIF-R		1.20 (0.66-2.16)	5.5E-01	1.31 (0.72-2.36)	3.8E-01
FGF-21		1.13 (0.96-1.34)	1.5E-01	1.13 (0.96-1.34)	1.4E-01
CCL19		0.97 (0.76-1.22)	7.8E-01	0.98 (0.77-1.24)	9.0E-01
IL-10RB		1.01 (0.62-1.62)	9.8E-01	1.02 (0.62-1.65)	9.4E-01
IL-18R1		1.69 (1.07-2.68)	2.4E-02	1.77 (1.13-2.79)	1.3E-02
CXCL5		1.03 (0.91-1.16)	6.5E-01	1.04 (0.92-1.17)	5.6E-01
TRANCE	x	0.44 (0.32-0.61)	1.3E-06	0.45 (0.33-0.61)	3.4E-07
HGF	x	1.75 (1.38-2.24)	5.0E-06	1.78 (1.39-2.29)	4.8E-06
IL-12B		0.81 (0.60-1.10)	1.9E-01	0.79 (0.58-1.07)	1.2E-01
MMP-10		0.75 (0.55-1.01)	6.3E-02	0.84 (0.62-1.12)	2.5E-01
IL-10	x	1.45 (1.09-1.96)	1.2E-02	1.61 (1.23-2.14)	6.4E-04
CCL23	x	2.84 (1.78-4.61)	1.7E-05	2.67 (1.7-4.24)	2.4E-05
CD5		0.48 (0.27-0.82)	8.1E-03	0.46 (0.26-0.79)	5.7E-03
CCL3		1.47 (0.99-2.16)	5.4E-02	1.53 (1.03-2.27)	3.3E-02
Flt3L	x	0.39 (0.25-0.59)	1.2E-05	0.39 (0.25-0.60)	1.9E-05
CXCL6		1.22 (0.93-1.59)	1.6E-01	1.17 (0.89-1.53)	2.6E-01
CXCL10		1.28 (1.03-1.59)	2.8E-02	1.32 (1.06-1.65)	1.1E-02
4E-BP1	x	1.39 (1.19-1.64)	4.4E-05	1.33 (1.14-1.57)	4.6E-04
SIRT2	x	1.37 (1.18-1.59)	2.4E-05	1.32 (1.15-1.53)	1.4E-04
CCL28		1.55 (1.01-2.34)	4.0E-02	1.47 (0.95-2.24)	7.7E-02
DNER	x	0.24 (0.12-0.47)	4.3E-05	0.25 (0.12-0.49)	6.0E-05
EN-RAGE	x	2.13 (1.69-2.72)	2.7E-10	2.09 (1.66-2.67)	8.6E-10
CD40		1.82 (1.14-2.91)	1.2E-02	1.56 (1.00-2.42)	5.0E-02
IFN-gamma		0.66 (0.39-1.04)	9.7E-02	0.72 (0.44-1.10)	1.6E-01
FGF-19		1.37 (1.09-1.73)	6.9E-03	1.22 (0.97-1.53)	9.7E-02
MCP-2		1.06 (0.76-1.48)	7.4E-01	0.95 (0.69-1.32)	7.8E-01
CASP-8	x	1.80 (1.3-2.51)	4.2E-04	1.75 (1.27-2.43)	6.9E-04
CCL25	x	0.48 (0.34-0.67)	2.6E-05	0.46 (0.32-0.64)	1.2E-05
CX3CL1		1.18 (0.74-1.84)	4.8E-01	1.27 (0.81-2.00)	2.9E-01
TNFRSF9		0.63 (0.4-0.96)	3.5E-02	0.6 (0.38-0.93)	2.4E-02
NT-3		1.16 (0.78-1.68)	4.5E-01	1.10 (0.73-1.60)	6.3E-01
TWEAK		0.67 (0.42-1.04)	8.2E-02	0.6 (0.37-0.94)	2.9E-02
CCL20		1.28 (1.04-1.57)	1.8E-02	1.30 (1.06-1.58)	1.0E-02
STAMPB	x	1.49 (1.23-1.82)	6.0E-05	1.38 (1.14-1.67)	8.9E-04
ADA		1.40 (0.90-2.19)	1.3E-01	1.32 (0.87-1.97)	1.8E-01
TNFB		0.59 (0.40-0.88)	9.8E-03	0.57 (0.38-0.84)	5.4E-03
CSF-1	x	4.44 (2.32-8.65)	8.4E-06	5.04 (2.67-9.82)	1.1E-06

OR<1 and *P*<0.05	OR >1 and *P*<0.05	*P*>0.05

Table S5, Continued:

Protein Name	FDR significant All_Univariate	Lipid lowering excluded (univariate)		Dead excluded (univariate)	
		Odds ratio	*P*-value	Odds ratio	*P*-value
IL-8		1.51 (1.16-1.99)	2.4E-03	1.40 (1.06-1.83)	1.5E-02
VEGF-A		2.00 (1.26-3.18)	3.4E-03	1.89 (1.16-3.07)	1.0E-02
MCP-3	x	2.03 (1.49-2.81)	1.2E-05	2.04 (1.49-2.81)	9.4E-06
GDNF		2.25 (1.38-3.73)	1.3E-03	2.03 (1.27-3.30)	3.6E-03
CDCP1		1.34 (0.95-1.88)	9.8E-02	1.43 (1.02-2.00)	4.0E-02
CD244		0.91 (0.50-1.64)	7.6E-01	1.25 (0.70-2.24)	4.5E-01
IL-7		1.48 (1.11-1.97)	6.9E-03	1.34 (1.01-1.77)	3.9E-02
OPG		1.62 (0.95-2.74)	7.5E-02	1.47 (0.86-2.49)	1.5E-01
LAP TGF-beta-1		1.56 (1.06-2.30)	2.4E-02	1.50 (1.00-2.24)	4.6E-02
uPA		0.39 (0.21-0.71)	2.8E-03	0.46 (0.25-0.85)	1.4E-02
IL-6	x	1.90 (1.57-2.33)	2.5E-10	2.06 (1.67-2.58)	8.2E-11
MCP-1		1.08 (0.70-1.67)	7.3E-01	1.06 (0.68-1.63)	7.9E-01
CXCL11		1.06 (0.86-1.31)	5.6E-01	1.08 (0.86-1.33)	5.1E-01
AXIN1		1.28 (1.08-1.51)	3.8E-03	1.33 (1.12-1.58)	9.7E-04
TRAIL	x	0.19 (0.11-0.33)	5.2E-09	0.22 (0.12-0.39)	2.4E-07
CXCL9		0.90 (0.70-1.14)	3.9E-01	1.00 (0.79-1.25)	9.7E-01
CST5		0.63 (0.39-1.00)	5.3E-02	0.57 (0.36-0.90)	1.8E-02
OSM	x	2.10 (1.67-2.66)	3.6E-10	1.89 (1.52-2.38)	2.5E-08
CXCL1		1.18 (0.98-1.42)	9.2E-02	1.11 (0.93-1.34)	2.6E-01
CCL4		1.56 (1.11-2.21)	1.1E-02	1.49 (1.07-2.08)	1.9E-02
CD6	x	0.51 (0.33-0.79)	2.4E-03	0.62 (0.40-0.94)	2.5E-02
SCF	x	0.49 (0.33-0.73)	3.9E-04	0.53 (0.36-0.80)	2.2E-03
IL-18		0.99 (0.68-1.43)	9.5E-01	1.11 (0.77-1.59)	5.7E-01
SLAMF1		1.29 (0.87-1.90)	1.9E-01	1.34 (0.91-1.96)	1.3E-01
MCP-4		0.87 (0.61-1.20)	4.0E-01	0.94 (0.67-1.30)	7.0E-01
CCL11		0.60 (0.38-0.91)	1.9E-02	0.74 (0.49-1.12)	1.6E-01
TNFSF14	x	4.11 (2.67-6.54)	5.7E-10	4.02 (2.57-6.45)	2.9E-09
FGF-23		0.92 (0.65-1.28)	6.4E-01	0.88 (0.62-1.23)	4.7E-01
LIF-R		1.23 (0.67-2.25)	5.0E-01	1.11 (0.61-2.01)	7.4E-01
FGF-21		1.11 (0.93-1.32)	2.5E-01	1.12 (0.95-1.33)	1.9E-01
CCL19		0.97 (0.76-1.22)	7.8E-01	0.98 (0.77-1.25)	9.0E-01
IL-10RB		0.90 (0.54-1.49)	6.9E-01	1.02 (0.63-1.66)	9.2E-01
IL-18R1		2.02 (1.26-3.25)	3.5E-03	1.66 (1.04-2.65)	3.3E-02
CXCL5		1.05 (0.92-1.19)	4.8E-01	1.01 (0.90-1.15)	8.2E-01
TRANCE	x	0.43 (0.31-0.59)	1.7E-07	0.46 (0.33-0.63)	3.5E-06
HGF	x	1.90 (1.48-2.45)	6.4E-07	1.67 (1.31-2.14)	3.8E-05
IL-12B		0.73 (0.53-1.00)	5.3E-02	0.86 (0.63-1.17)	3.3E-01
MMP-10		0.84 (0.62-1.13)	2.7E-01	0.71 (0.51-0.96)	3.1E-02
IL-10	x	1.63 (1.18-2.26)	3.3E-03	1.47 (1.10-1.99)	1.1E-02
CCL23	x	2.95 (1.87-4.74)	4.9E-06	2.70 (1.68-4.39)	4.7E-05
CD5		0.38 (0.21-0.68)	1.4E-03	0.51 (0.29-0.88)	1.8E-02
CCL3		1.58 (1.06-2.37)	2.5E-02	1.51 (1.02-2.24)	4.0E-02
Flt3L	x	0.38 (0.24-0.58)	1.3E-05	0.42 (0.27-0.63)	5.0E-05
CXCL6		1.16 (0.88-1.53)	3.0E-01	1.19 (0.91-1.57)	2.1E-01
CXCL10		1.22 (0.97-1.54)	8.3E-02	1.29 (1.03-1.62)	2.3E-02
4E-BP1	x	1.37 (1.16-1.62)	1.7E-04	1.36 (1.16-1.61)	1.6E-04
SIRT2	x	1.36 (1.17-1.58)	5.5E-05	1.36 (1.18-1.58)	3.9E-05
CCL28		1.32 (0.85-2.01)	2.0E-01	1.50 (0.98-2.29)	6.0E-02
DNER	x	0.21 (0.10-0.43)	1.7E-05	0.24 (0.12-0.48)	6.7E-05
EN-RAGE	x	2.12 (1.68-2.72)	9.1E-10	2.10 (1.66-2.68)	9.6E-10
CD40		1.56 (0.98-2.47)	5.7E-02	1.82 (1.13-2.93)	1.3E-02
IFN-gamma		0.62 (0.35-1.01)	7.5E-02	0.65 (0.38-1.04)	9.2E-02
FGF-19		1.24 (0.98-1.58)	7.3E-02	1.41 (1.12-1.79)	4.3E-03
MCP-2		0.91 (0.65-1.29)	6.0E-01	1.08 (0.77-1.52)	6.4E-01
CASP-8	x	1.76 (1.27-2.46)	7.0E-04	1.74 (1.25-2.43)	1.1E-03
CCL25	x	0.40 (0.27-0.57)	6.5E-07	0.48 (0.33-0.67)	3.6E-05
CX3CL1		0.98 (0.60-1.59)	9.4E-01	1.17 (0.74-1.85)	4.9E-01
TNFRSF9		0.55 (0.34-0.86)	1.1E-02	0.67 (0.43-1.04)	7.7E-02
NT-3		1.17 (0.75-1.79)	4.6E-01	1.11 (0.74-1.63)	5.8E-01
TWEAK		0.60 (0.37-0.96)	3.5E-02	0.63 (0.39-0.99)	5.1E-02
CCL20		1.28 (1.04-1.57)	1.8E-02	1.27 (1.03-1.56)	2.1E-02
STAMPB	x	1.44 (1.18-1.76)	2.7E-04	1.49 (1.22-1.81)	8.8E-05
ADA		1.48 (0.98-2.24)	6.1E-02	1.37 (0.86-2.15)	1.7E-01
TNFB		0.55 (0.37-0.83)	4.6E-03	0.61 (0.40-0.90)	1.5E-02
CSF-1	x	5.53 (2.80-11.2)	1.3E-06	4.16 (2.16-8.15)	2.5E-05

OR<1 and $P<0.05$	OR >1 and $P<0.05$	$P>0.05$
